# Sparse testcrossing for early-stage genomic prediction of general combining ability to increase genetic gain in maize hybrid breeding programs

**DOI:** 10.1007/s00122-026-05169-x

**Published:** 2026-02-24

**Authors:** David O. González-Diéguez, Gary N. Atlin, Yoseph Beyene, Dagne Wegary, Dorcus C. Gemenet, Christian R. Werner

**Affiliations:** 1 Breeding for Tomorrow (B4T) Accelerate, Consultative Group of International Agricultural Research (CGIAR), 56237 Texcoco, Mexico; 2 International Maize and Wheat Improvement Center (CIMMYT), Texcoco, Mexico; 3 International Maize and Wheat Improvement Center (CIMMYT), Nairobi, Kenya; 4 International Maize and Wheat Improvement Center (CIMMYT), Harare, Zimbabwe; 5 Bill & Melinda Gates Foundation, Seattle, WA 98109 USA

## Abstract

**Key message:**

**Sparse testcrossing with 3-5 testers enhances genetic gain in hybrid breeding programs**,** offering a practical balance of simple testcross designs**,** resource efficiency**,** and increased prediction accuracy for general combining ability.**

**Abstract:**

Sparse testcrossing is an effective strategy for increasing both short- and long-term genetic gain in hybrid breeding programs. Maize hybrid breeding programs aim to develop new hybrid varieties by crossing genetically distinct parents from different heterotic pools, exploiting heterosis for improved performance. The programs typically consist of two main components: population improvement and product development. The population improvement component aims to enhance the heterotic pools through reciprocal recurrent selection based on general combining ability (GCA). However, especially in the early stages of testing, evaluating large numbers of hybrid combinations to estimate GCA is impractical due to considerable logistical challenges and costs. Therefore, breeders often evaluate the initial population of selection candidates using only a single tester to narrow down the candidate pool before further evaluation. Using a single tester, however, may not adequately represent the heterotic pool, leading to inaccurate GCA estimates and suboptimal selection decisions. To address this, we propose sparse testcrossing for early-stage testing, where subsets of candidate genotypes are testcrossed with different testers, connected through a genomic relationship matrix. We conducted stochastic simulations to compare various sparse testcrossing designs with a conventional testcross strategy using a single tester over 15 cycles of reciprocal recurrent genomic selection. Our results show that using 3–5 testers, sparsely distributed among full-sibs, sparse testcrossing offers breeders a practical balance between simple testcross designs, resource efficiency, and increased prediction accuracy for GCA, ultimately resulting in increased rates of genetic gain.

**Supplementary Information:**

The online version contains supplementary material available at 10.1007/s00122-026-05169-x.

## Introduction

Sparse testcrossing is an effective strategy for increasing both short- and long-term genetic gain in hybrid breeding programs. By using 3–5 testers, sparsely distributed among full-sibs, it offers breeders a practical balance between simple testcross designs, resource efficiency, and increased prediction accuracy for general combining ability (GCA) compared to conventional testcross strategies using a single tester. As such, sparse testcrossing lays the foundation for rapid recycling of parents at the earliest stage of agronomic testing to minimize breeding cycle time and maximize genetic gain per unit of time.

Maize hybrid breeding programs aim to develop high-performing hybrid varieties by crossing genetically distinct parental genotypes, typically derived from different populations referred to as heterotic pools. In this way, hybrid breeding exploits heterosis (hybrid vigor), which results from the combination of beneficial complementary haplotypes accumulated in these heterotic pools, leading to improved performance in the hybrids compared to their parents (Melchinger and Gumber 1998). Hybrids can be produced through various crossing strategies, including single cross (crossing two inbred lines), three-way cross (crossing a single cross with a third inbred line), or double cross hybrids (crossing two single crosses) (Bernardo 2020). While many commercial programs with a long history of hybrid maize breeding typically focus on single-cross hybrids, public sector programs and small to medium-sized seed companies often use three-way crosses, with the maternal component being a single-cross hybrid, to compensate for low seed set caused by inbreeding depression (Collinson et al. 2022; Mukaro et al. 2024).

Hybrid breeding programs typically consist of two key components: (1) population improvement and (2) product development (Gaynor et al. 2017; Powell et al. 2020). The population improvement component aims to enhance the genetic composition of the heterotic pools for both per se performance and complementarity between the pools, thereby creating superior hybrid parents. Population improvement is achieved through reciprocal recurrent selection of genotypes, such as inbred lines, based on GCA (Comstock et al. 1949). The GCA predicts the average performance of a genotype when randomly crossed with genotypes from the complementary heterotic pool (Hallauer et al. 2010). It reflects both additive genetic effects and, to some extent, non-additive effects that arise from complementary haploblock interactions, and serves as a predictor of a parent’s ability to produce high-performing hybrids when crossed with other genotypes from a complementary heterotic pool (Bernardo 2020). Following population improvement, the product development component then centers on identifying the best-performing hybrid combinations among all potential reciprocal crosses between parental genotypes from the improved heterotic pools.

Ideally, GCA is estimated using a complete factorial mating design, which evaluates all possible hybrid combinations between heterotic pools (Comstock and Robinson 1948; Fritsche-Neto et al. 2018). However, the implementation of such a mating design at early stages of testing is often impractical due to the large number of hybrid combinations and the associated logistical challenges and costs. To address this, maize breeders typically use one or a few testers, selected to represent the complementary heterotic pool (Hallauer et al. 2010).

In many public maize hybrid breeding programs, it is common practice to employ a stage-wise selection approach before parents are recycled. First, the initial population of candidate genotypes is typically evaluated using a single tester, providing a preliminary assessment to narrow down the candidate pool. Then, the pre-selected subset of genotypes is further evaluated using additional testers—usually 3 to 5—to enhance GCA accuracy, as more testers better capture the genetic diversity and haploblock frequencies in the complementary heterotic pool. However, this approach comes at the cost of extending the breeding cycle time and slowing genetic gain. Additionally, pre-selection with a single tester in the first stage may not adequately represent the complementary heterotic pool, leading to inaccurate GCA estimates and suboptimal advancement of candidates to subsequent testing stages.

Genomic selection offers great potential to increase GCA accuracy in the first stage of testing. By leveraging haploblocks as the primary units of evaluation rather than individual genotypes (Bingham 1998; Meuwissen et al. 2001), genomic selection enhances prediction accuracy and can reduce breeding cycle time, thereby accelerating genetic gain (Crossa et al. 2017). Genomic selection has already proven to be a valuable tool to improve maize hybrid breeding programs, particularly for predicting hybrid performance (Technow et al. 2014; Kadam et al. 2016; Fritsche-Neto et al. 2018; Seye et al. 2020) and for predicting GCA for parent recycling (Melchinger and Frisch 2023; de Jong et al. 2023; Lorenzi et al. 2024). Several studies on genomic prediction of GCA suggest that incomplete factorial testcross designs improve GCA prediction accuracy and genetic gain compared to single-tester designs (Melchinger and Frisch 2023; Lorenzi et al. 2024). However, incomplete factorial designs require labor-intensive hand pollination, which is costly and logistically challenging, particularly for synchronizing flowering time. In contrast, conventional testcross approaches with a single tester often use open pollination for producing testcrosses, offering simple logistics but resulting in low GCA accuracy (Fritsche-Neto et al. 2018). Therefore, there is a need for testcross designs that retain much of the logistical simplicity of single-tester designs while recovering the GCA prediction accuracy of incomplete factorials.

In this study, we propose sparse testcrossing for early-stage testing in hybrid breeding programs as an alternative to conventional testcross designs with a single tester. By exploiting genomic relationship information, sparse testcrossing connects candidate genotypes from one heterotic pool to multiple testers from a complementary heterotic pool. This strategy builds on the idea of sparse testing (Endelman et al. 2014; Werner et al. 2025), conceptually treating testers as distinct “testing environments”. Although each candidate genotype is crossed to only a single tester, sets of related genotypes that share haplotypes are crossed to various testers, increasing the coverage of haplotype diversity in the complementary tester pool. As a result, the genomic predictions of GCA for the haplotypes carried by a genotype reflect their value across multiple testcrosses rather than being tied to a single tester.

We hypothesize that sparse testcrossing can substantially increase GCA prediction accuracy in early stage testing of hybrid breeding programs. To test our hypothesis, we conducted stochastic simulations comparing ten sparse testcrossing designs with a conventional single-tester design over 15 cycles of reciprocal recurrent selection, representing the population improvement component of a rapid cycle genomic selection hybrid breeding program. Our results indicate that, when using 3–5 testers sparsely allocated among full sibs, sparse testcrossing provides a favorable compromise between design simplicity, resource efficiency and GCA prediction accuracy, leading to higher rates of genetic gain and enabling early-stage parent selection without increasing testing resources. Furthermore, our results are not limited to inbred-hybrid breeding in maize, but bear generality for other diploid(-like) inbred-hybrid crops, such as crops with a much shorter hybrid breeding history, such as canola or sorghum.

## Materials and methods

Stochastic simulations were used to compare ten different early-stage sparse testcrossing designs for genomic prediction of general combining ability (GCA) in hybrid breeding programs. The sparse testcrossing designs were evaluated for prediction accuracy of GCA and hybrid genetic gain over 15 cycles of reciprocal recurrent genomic selection and compared to a conventional early-stage testcross strategy with a single tester. Comparisons were based on a single quantitative trait, such as yield, under three different dominance degrees.

The material and methods are subdivided into five sections:

(i) Simulation of the founder population and heterotic pool formation
(ii) Description of the sparse testcrossing designs
(iii) Simulation of 15 cycles of reciprocal recurrent genomic selection
(iv) Evaluation of sparse testcrossing designs for GCA prediction accuracy and genetic gain
(v) Description of the genomic prediction models

### Simulation of the founder population and heterotic pool formation

#### Genome simulation

Whole-genome sequences were simulated for a founder population of 80 heterozygous genotypes. To roughly mimic the maize genome, each founder genome consisted of 10 chromosome pairs with a physical length of 2 × 10^8^ base pairs (bp) and a genetic length of 200 centimorgans (cM), resulting in a total physical length of 2 Gbp and a genetic length of 2,000 cM. Whole-genome sequences were generated using the Markovian coalescent simulator (Chen et al. 2009) in AlphaSimR (Gaynor et al. 2017), deploying AlphaSimR’s maize evolutionary history default settings. Recombination rate was derived as the ratio between genetic length and physical length (i.e., 2 morgans/2 × 10^8^ bp = 10^–8^ recombinations per base pair), and mutation rate was set to 1.25 × 10^–8^ per base pair. The effective population size (Ne) at the end of the coalescent process was set to 100. The main purpose of the coalescence process was to generate linkage disequilibrium in the founder population.

A set of 500 biallelic quantitative trait nucleotides (QTN) and 500 single nucleotide polymorphisms (SNP) were randomly sampled along each chromosome to simulate a quantitative trait controlled by 5,000 QTN and a SNP marker array with 5,000 markers. Founder genotypes were formed by randomly sampling 10 chromosome pairs per genotype.

#### Simulation of genetic values

Genetic values for a single quantitative trait were simulated by summing biological additive genetic effects and dominance effects at the 5,000 QTN. Additive genetic effects (*a*) were randomly sampled from a standard normal distribution and scaled to obtain an additive genetic variance in the founder population of ${\sigma }_{A}^{2}=20$, following a study by De Jong et al. (2023) on the comparison of genomic prediction models for GCA.

Dominance effects ($d$) were calculated by multiplying the absolute biological additive effect of a QTN (${a}_{i}$) by a locus-specific dominance degree (${\delta }_{i}$), that is ${d}_{i}={\delta }_{i}\times \left|{a}_{i}\right|$. Dominance degrees were sampled from a normal distribution with mean dominance coefficient ${\mu }_{\delta }$ and variance ${\sigma }_{\delta }^{2}$, that is ${\delta }_{i}\sim N\left({\mu }_{\delta },{\sigma }_{\delta }^{2}\right)$. Three levels of average dominance degrees (0.1, 0.5, and 0.9) were used, each representing a separate simulation scenario of overall positive directional dominance. The dominance degree variance ${\sigma }_{\delta }^{2}$ was set to 0.2. This resulted, on average, in 2%, 13% and 41% of loci exhibiting overdominance. Under these settings, the SCA to GCA variance ratios in the first breeding cycle were 0.05, 0.23, and 0.50 for low (${\mu }_{\delta }$=0.1), medium (${\mu }_{\delta }$=0.5) and high (${\mu }_{\delta }$=0.9) dominance degrees, respectively.

#### Formation of initial heterotic pools

**Fig. 1:**
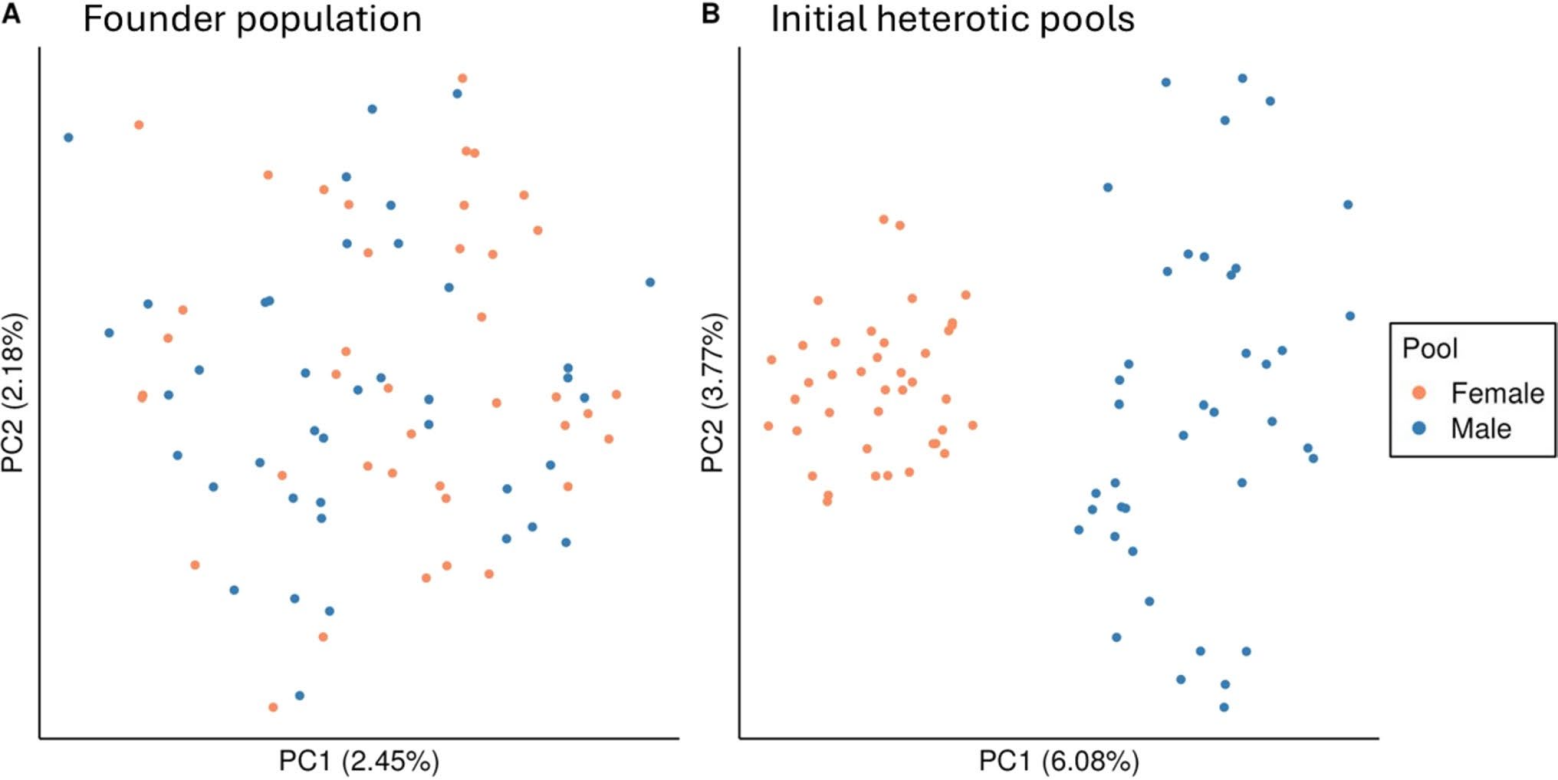
Principal component analysis (PCA) of the genomic relationship matrix of the founder population before **A** and after **B** three generations of random crossing and selection to generate baseline family structure within pools. The 80 founder genotypes were randomly assigned to the two heterotic pools. Orange and blue points represent the female and male heterotic pools, respectively. The first and second principal components (PC1 and PC2) are shown on the x and y axes, respectively. The proportion of variance explained by PC1 and PC2 is given in parentheses. Results shown correspond to a randomly selected simulation replicate under medium dominance degree

The 80 founder genotypes were converted into homozygous inbred lines and randomly allocated to one of two pools of 40 inbred lines each (Fig. 1A). The two pools served as separate male and female heterotic pools. To create a baseline family structure within heterotic pools as a basis for genomic prediction, three generations of random crossing and selection were conducted. At first, 60 bi-parental crosses were sampled from a half diallel without selfing to produce F_1_ genotypes, ensuring that each of the 40 inbred lines was used in at least one cross. Then, 20 fully homozygous inbred lines were created from each F_1_, resulting in a total of 1,200 inbred lines per heterotic pool. Among those inbred lines, 40 lines were randomly selected as new parents. This procedure was repeated for two more generations, resulting in appreciable genetic differentiation between pools (Fig. 1B).

### Description of the sparse testcrossing designs

We compared ten sparse testcrossing designs employing 2, 3, 4, 5, 10, 20, 50, 100, 600, and 1,200 testers, respectively, to generate 1,200 testcrosses per heterotic pool (Table 1). Each tester was crossed with an equally sized subset of inbred lines randomly sampled from the other heterotic pool, allowing full-sibs lines (i.e. lines derived from the same bi-parental cross) to be crossed with different testers. A conventional early-stage testcross design with a single tester crossed to all 1,200 inbred lines served as benchmark scenario. At each cycle, new testers for both heterotic pools were selected at random among inbred lines in that pool to avoid confounding effects between the testcross strategy and the tester selection strategy.

**Table 1:** Number of testers and testcrosses per tester in the conventional early-stage testcross design with a single tester (benchmark; first column) and the ten sparse testcrossing designs

# Testers	1	2	3	4	5	10	20	50	100	600	1200
# Crosses per Tester	1,200	600	400	300	240	120	60	24	12	2	1

### Simulation of 15 cycles of reciprocal recurrent genomic selection

**Fig. 2:**
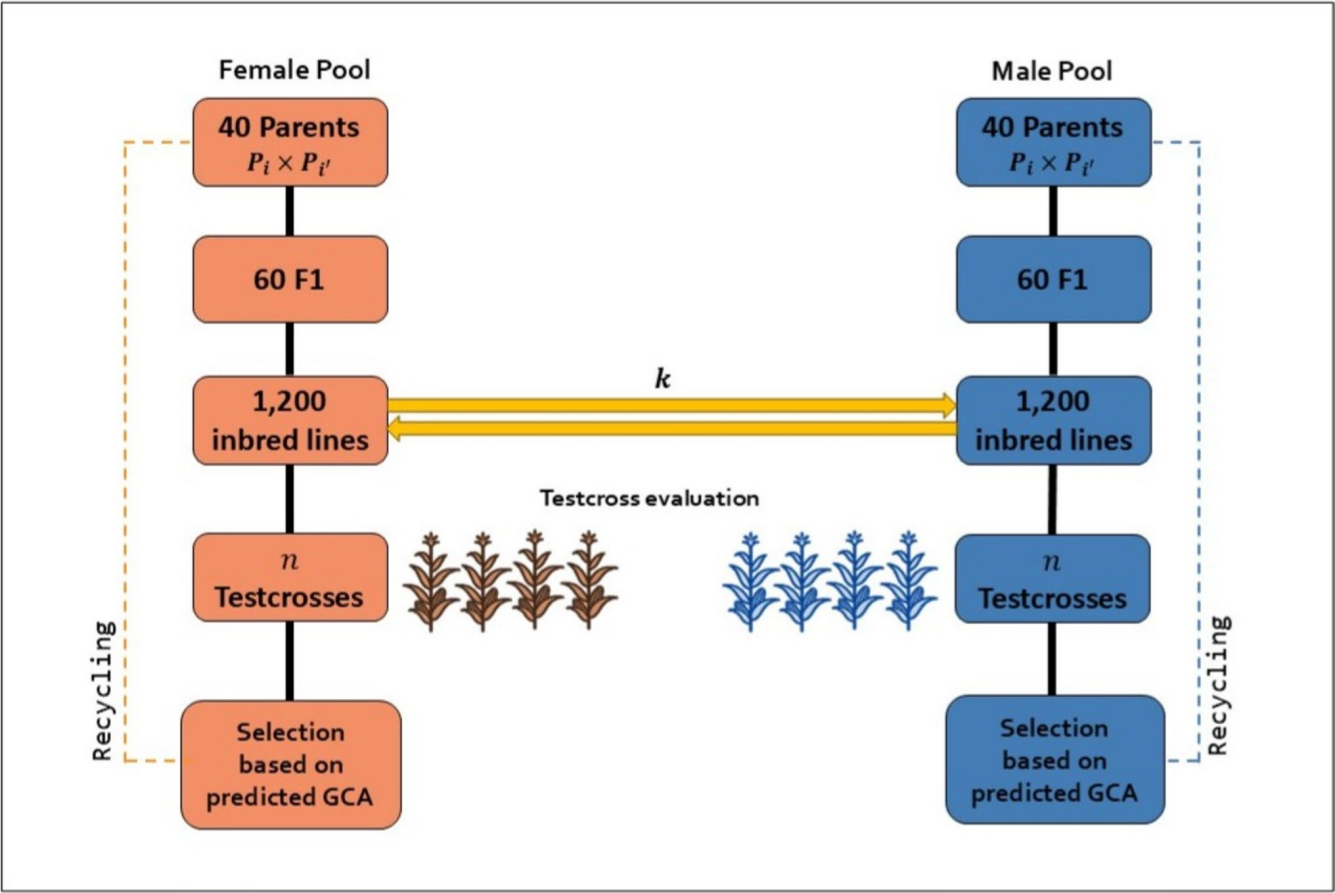
Schematic representation of the simulated hybrid breeding scheme, where $n$ denotes the number of testcrosses, and $k$ the number of testers. This scheme was used both as the baseline breeding program for comparing GCA prediction accuracy among testcrossing designs and as the template for running separate programs for each testcrossing strategy to assess hybrid genetic gain

To compare the ten sparse testcrossing designs and the conventional early-stage testcross design with a single tester for genomic prediction accuracy of GCA and hybrid genetic gain, we simulated 15 cycles of reciprocal recurrent genomic selection, representing the population improvement component of a rapid cycle genomic selection hybrid breeding program with inbred lines. The basic structure of the breeding pipeline is shown in Fig. 2 and hereinafter described for one heterotic pool.

At each breeding cycle, 60 bi-parental crosses were sampled from a half diallel without selfing between the 40 inbred lines serving as parents to generate F_1_ genotypes. Then, from each F_1_, 20 homozygous inbred lines were derived, resulting in a total of 1,200 inbred lines. These inbred lines were testcrossed with the testers from the other heterotic pool.

Testcross phenotypes were generated by adding a random error to the testcross genotypic values. The random error was sampled from a normal distribution with mean zero and the error variance ${\sigma }_{e}^{2}$ was set to obtain a broad-sense heritability of *H*^*2*^ = 0.3 in the hypothetical population of all 40 × 40 = 1,600 possible hybrids between the two heterotic pools (i.e., ${\sigma }_{e}^{2}={\sigma }_{G}^{2}\left(1-{H}^{2}\right)/{H}^{2}$, with ${\sigma }_{G}^{2}$ being the genetic variance of all possible hybrids). The error variance ${\sigma }_{e}^{2}$ was kept constant across all 15 breeding cycles. The 40 inbred lines with the highest predicted GCA were selected as new parents.

### Evaluation of sparse testcrossing designs for GCA prediction accuracy and hybrid genetic gain

Two different simulation approaches were used to compare the sparse testcrossing designs, one addressing genomic prediction accuracy of GCA and the other one addressing hybrid genetic gain. Each simulation was replicated 100 times.

#### Evaluation of hybrid genetic gain

Hybrid genetic gain was evaluated by simulating the rapid cycle genomic selection hybrid breeding program (Fig. 2) separately for each of the ten sparse testcrossing designs and for the conventional single-tester design. At each cycle, new parents were selected based on genomic predicted GCA calculated with the “Testcross Model” (Eq. 1) for the conventional single-tester design and the “Hybrid Pool Specific Model” (see Eq. 2) for the sparse testcross designs. Genetic gain was measured as the mean total genotypic value of all 1,440,000 potential hybrids from a full factorial between the two heterotic pools and expressed relative to the mean genetic value of all possible hybrids formed between initial heterotic pools (Fig. 1B).

This simulation approach allowed direct comparison of testcross designs for genetic gain over breeding cycles. However, because each design generated its own selection trajectory and pattern of genetic variance (Suppl. Fig. S12), it is not suitable for direct comparison of GCA prediction accuracies (Suppl. Fig. S14).

#### Evaluation of GCA prediction accuracy

Prediction accuracy of GCA was evaluated by simulating a baseline hybrid breeding program in which all 1,200 inbred lines were testcrossed with the same three testers, resulting in 3 × 1,200 = 3,600 testcrosses per heterotic pool. At each cycle, new parents were selected based on genomic predicted GCA calculated with the “Testcross Model” (Eq. 1), using the average testcross performance of inbred lines. This simulation approach generated a single selection trajectory, starting from an emerging hybrid breeding program with low between pool divergence to a well-established program with high between-pool divergence. The baseline breeding program mimics the population improvement component of a conventional reciprocal recurrent genomic selection hybrid breeding program (Fig. 2), in which inbred lines are usually crossed to and evaluated with several testers before new parents are identified.

At cycles 1, 2, 3, 5, 10, and 15 of the baseline breeding program, we applied the ten sparse testcrossing designs and the single-tester design to the same set of inbred lines and testers to evaluate genomic prediction accuracy of GCA. This enabled comparison of the testcrossing strategies across different stages of genetic divergence and diversity. Prediction accuracy was calculated as the Pearson correlation coefficient between genomic predicted GCA and true GCA. Genomic predicted GCA was calculated using the “Testcross Model” (Eq. 1) for the conventional single-tester design and the “Hybrid Pool Specific Model” (see Eq. 2) for the sparse testcross designs. The true GCA was calculated as the mean genetic value of an inbred line from one heterotic pool crossed with all inbred lines from the other heterotic pool. Phenotypic GCA prediction accuracy was calculated as the correlation between the testcross phenotype and the true GCA. Because each line was crossed with only one tester, phenotypic prediction relied on a single observation per line.

Because the baseline program used three times as many testcrosses as the compared testcross strategies and served only to provide a shared population history so that all testcross designs were compared under identical conditions, it was not included in the evaluations.

Overall, this simulation approach aims to provide real-world hybrid breeding programs with general, broadly applicable trends that allow them to map their own level of heterotic pool differentiation onto our simulation results and draw conclusions about suitable numbers of testers.

#### Divergence of heterotic pools, genetic variance components and diversity

Divergence of heterotic pools over breeding cycles was monitored using three metrics: (1) Nei’s minimum genetic distance between the pools, (2) the average heterosis observed between the two pools, and (3) the fraction of fixed alleles in the two pools. Nei’s minimum genetic distance was calculated as ${D}_{female,male}=\frac{1}{n}\sum_{i=1}^{n}{\left({p}_{i}^{female}-{p}_{i}^{male}\right)}^{2}$ (Nei 1987)**,** where *n* is the number of polymorphic QTN, and ${p}_{i}^{female}$ and ${p}_{i}^{male}$ are the allele frequencies in the female and male heterotic pool at the ${i}^{th}$ QTN, respectively. Mid-parent heterosis, defined as the deviation of hybrid performance from the mean of the two parental populations, was calculated as $H={\sum_{i=1}^{n}{d}_{i}\left({p}_{i}^{female}-{p}_{i}^{male}\right)}^{2}$ (Falconer 1981). The fraction of fixed alleles was calculated relative to the total number of simulated QTN, where QTN with allele frequencies of 0 in one pool and 1 in the other pool were considered fixed for opposite alleles, while QTN with allele frequency of either 0 or 1 in both pools were considered fixed for the same allele.

The total genetic variance of the hybrids was calculated as the variance of the true genetic values of all possible hybrids between the two heterotic pools. The GCA variance was calculated as the variance of the true GCA values of all inbred lines. The SCA variance was calculated as the difference between hybrid genetic variance and GCA variance.

### Genomic prediction models

Depending on the testcross design, two different models for genomic prediction of GCA were used. For the conventional single-tester designs, we used a “Testcross Model” (Eq. 1), which was fitted separately for each heterotic pool. This modeling approach is common in single-tester designs, where SCA effects cannot be distinguished from GCA effects. For the sparse testcross designs with more than one tester, we used a “Hybrid Pool Specific Model” (Eq. 2), which jointly predicted heterotic pool-specific GCA for the inbred lines and testers, and SCA for testcross hybrids.

#### Testcross model

For the conventional early-stage testcross design with a single-tester, we used a model which was fitted separately for each heterotic pool to predict GCA effects of SNP marker alleles (Eq. 1):

1 $$y = 1\mu + Z\alpha + e$$

where ${\boldsymbol{y}}$ is the $n\times 1$ vector of testcross phenotypes with $n$ being the number of testcrosses, $1$ is an $n\times 1$ vector of ones, $\mu$ is the intercept; ${\boldsymbol{Z}}$ is the $n\times m$ matrix of inbred line genotypes, with $m$ being the number of SNP markers coded as 0 and 2 for genotypes aa and AA; $\boldsymbol{\alpha }$ is an $m\times 1$ vector of average effects of SNP marker alleles capturing GCA; and ${\boldsymbol{e}}$ is the $n\times 1$ vector of residuals. The GCA of the inbred lines was then predicted by summing the products of the marker effects and the corresponding inbred line genotypes. This model is commonly used in single-tester designs, where SCA effects cannot be distinguished from GCA effects, and hereafter will be referred to as “Testcross Model” (De Jong et al. 2023). It was fitted using the *RRBLUP()* function in AlphaSimR.

#### *Hybrid pool specific additive effects* + *dominance model*

For the sparse testcross designs with more than one tester, we used a model which jointly predicted heterotic pool-specific GCA effects of SNP marker alleles for the inbred lines and testers, and dominance marker effects for the testcross hybrids (Eq. 2):

2 $${\boldsymbol{y}} = 1\mu + Z_{1} a_{1} + Z_{2} a_{2} + W1\mu_{d} + Wd^{*} + e$$

where ${\boldsymbol{y}}$ is the $n\times 1$ vector of testcross phenotypes with $n$ being the number of testcrosses, ${{\boldsymbol{Z}}}_{1}$ is the $n\times m$ genotype matrix of inbred lines from one heterotic pool with $m$ being the number of SNP markers coded as 0 and 1 for genotypes aa and AA; ${{\boldsymbol{Z}}}_{2}$ is the $n\times m$ genotype matrix of the testers from the other heterotic pool, with genotypes coded as in ${{\boldsymbol{Z}}}_{1}$; ${{\boldsymbol{a}}}_{1}$ and ${{\boldsymbol{a}}}_{2}$ are the $m\times 1$ vectors of “biological” additive marker effects (Vitezica et al. 2013) for inbred lines and testers, respectively; ${\boldsymbol{W}}$ is an $n\times m$ dominance incidence matrix coded as 0, 1, and 0 for testcross genotypes AA, Aa, and aa, respectively; ${\boldsymbol{W}}1$ is an $n\times 1$ vector containing the total number of heterozygous loci for each testcross hybrid, and ${\mu }_{d}$ is the mean dominance effect across all loci, so that ${\boldsymbol{W}}1{\mu }_{d}$ results in an average of dominance effects for each individual testcross hybrid; ${{\boldsymbol{d}}}^{*}$ is an $m\times 1$ vector of “biological” dominance marker effects not captured by ${\boldsymbol{W}}1{\mu }_{d}$ (i.e., ${{\boldsymbol{d}}}^{*}={\boldsymbol{d}}-{\mu }_{d}$) with $E\left({{\boldsymbol{d}}}^{*} \right)=0$; and ${\boldsymbol{e}}$ is the $n\times 1$ vector of residuals. The model was fitted using the *RRBLUP_SCA()* function in AlphaSimR.

Pool-specific additive effects (${a}_{1}$ and ${a}_{2}$) and dominance effects ($d$) were then used to calculate the GCA effects of each SNP marker in heterotic pool 1 and 2 (${\widehat{\alpha }}_{1}$ and ${\widehat{\alpha }}_{2}$), respectively (Eq. 3):

3 $$\hat{\alpha }_{1} = \frac{1}{2}\left[ {\hat{a}_{1} + \hat{d}\left( {q_{2} - p_{2} } \right)} \right]\;{\mathrm{and}}\;\hat{\alpha }_{2} = \frac{1}{2}\left[ {\hat{a}_{2} + \hat{d}\left( {q_{1} - p_{1} } \right)} \right]$$

with ${p}_{1}$ and ${q}_{1}=1-{p}_{1}$ being the SNP marker allele frequencies in pool 1, and ${p}_{2}$ and ${q}_{2}$ being the SNP markers allele frequencies in pool 2 (González-Diéguez et al. 2021; De Jong et al. 2023). The GCA of the inbred lines was then predicted by summing the products of the marker effects and the corresponding inbred line genotypes. This was done using the *setEBV()* function in AlphaSimR. This model facilitates prediction of GCA and SCA effects while accounting for pool-specific LD between QTN and markers, as well as directional dominance, and hereafter will be referred to as “Hybrid Pool Specific Additive Effects + Dominance Model” (De Jong et al. 2023).

## Results

Our results show that sparse testcrossing can significantly improve genomic prediction accuracy of general combining ability (GCA) compared to a conventional early-stage testcross strategy using a single tester, thereby facilitating increased rates of hybrid genetic gain. Both genomic prediction accuracy of GCA and hybrid genetic gain increased with the number of testers until reaching a plateau around 10 testers. Sparse testcrossing improved prediction accuracy and genetic gain most during the early breeding cycles, while these advantages decreased as the genetic distance between pools increased. Furthermore, the benefits of sparse testcrossing were more pronounced in simulation scenarios with high dominance degrees but diminished as the dominance degree decreased.

Since both heterotic pools produced comparable outcomes, detailed results are presented only for one pool under medium dominance degree. Results for low and high dominance degrees are reported only when relevant and are otherwise provided in the supplementary material.

### Divergence of heterotic pools, genetic variances and diversity in the baseline breeding program

**Fig. 3:**
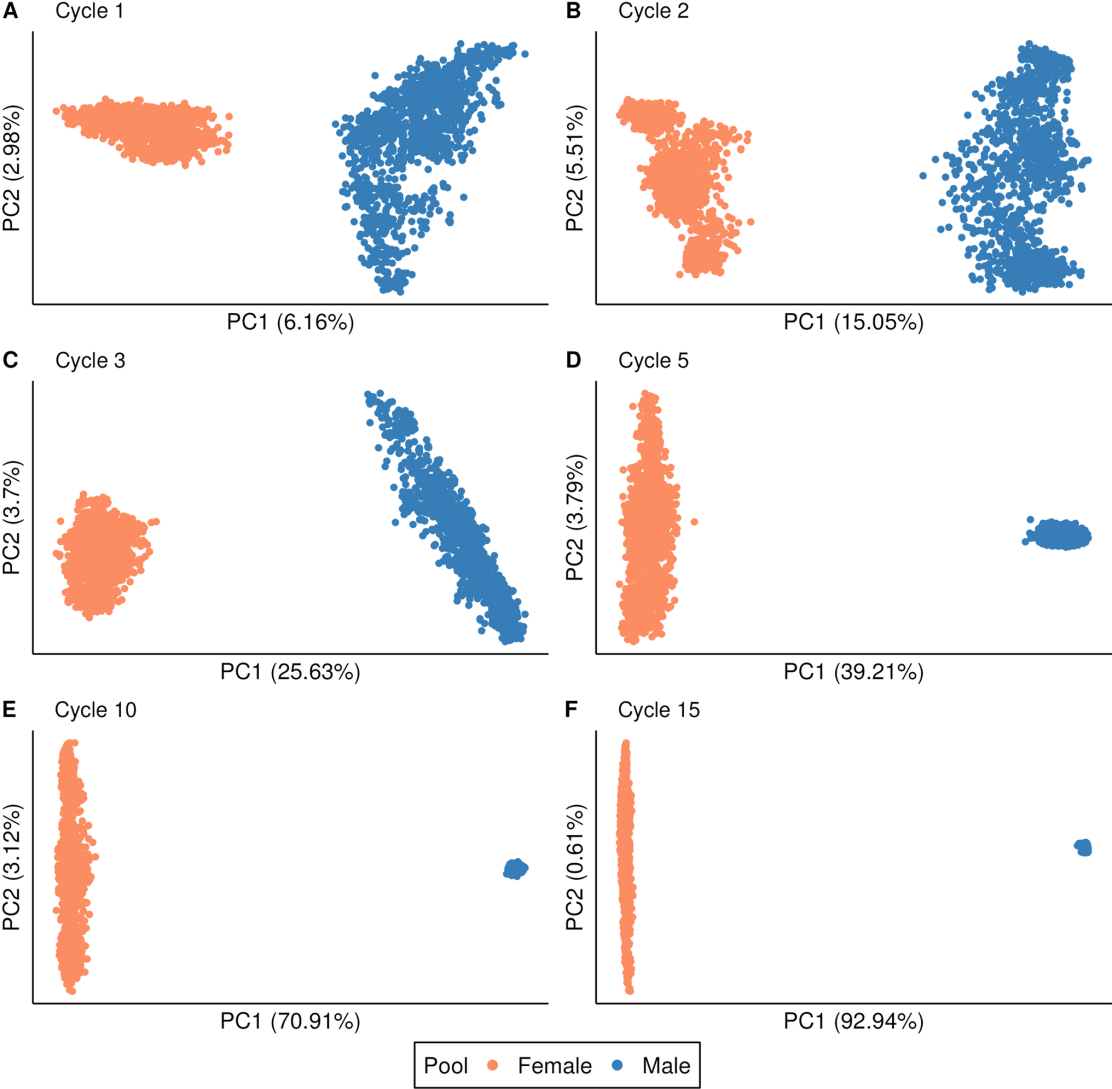
Principal component analysis (PCA) of the genomic relationship matrix of the two heterotic pools in the baseline breeding program at cycles 1, 2, 3, 5, 10, and 15. Orange and blue points represent the female and male heterotic pools, respectively. The first and second principal components (PC1 and PC2) are shown on the x- and y-axes, respectively. The proportion of variance explained by PC1 and PC2 is given in parentheses. Results shown correspond to a single, randomly selected simulation replicate of the baseline breeding program under medium dominance degree

As expected, selection of parents based on GCA led to divergence of the heterotic pools over time. This is shown in Fig. 3, which plots the first two principal components from a principal component analysis (PCA) of the genomic relationship matrix of the inbred lines at cycles 1, 2, 3, 5, 10, and 15 for a randomly selected simulation replicate of the baseline breeding program under medium dominance. At cycle 1, population structure was weak, with the first principal component (PC) explaining only 6.16% of the total variance (Fig. 3A). By cycle 15, the variance explained by the first PC had increased to 92.94% (Fig. 3F). Similar patterns of heterotic pool divergence were observed for the baseline breeding program under low and high dominance degrees (Suppl. Figs. S1 and S2).

Divergence of the heterotic pools was accompanied by an increase in genetic distance between pools (Suppl. Fig. S3A), an increase in heterosis (Suppl. Fig. S3B), and a higher fraction of fixed alleles in both pools (Suppl. Fig. S3C). The dominance degree differentially affected fixation rates of same versus opposite alleles: opposite alleles fixed faster under high dominance, whereas same alleles fixed faster under low dominance. This reflects the higher proportion of loci exhibiting overdominance under high dominance degree (41%) compared with low dominance degree (2%). Because divergence between pools is primarily driven by fixation of opposite alleles, higher dominance degrees led to faster and more pronounced differentiation between the heterotic pools. With ongoing selection, hybrid genetic variance, GCA variance and SCA variance decreased over breeding cycles (Suppl. Fig. S4). This reduction was strongest in the high dominance degree scenario, which after 15 cycles showed greater genetic distance and heterosis between pools (Suppl. Fig. S3A,B), along with a stronger reduction in both GCA and SCA variances (Suppl. Fig. S4).

### Prediction accuracy of general combining ability

**Fig. 4:**
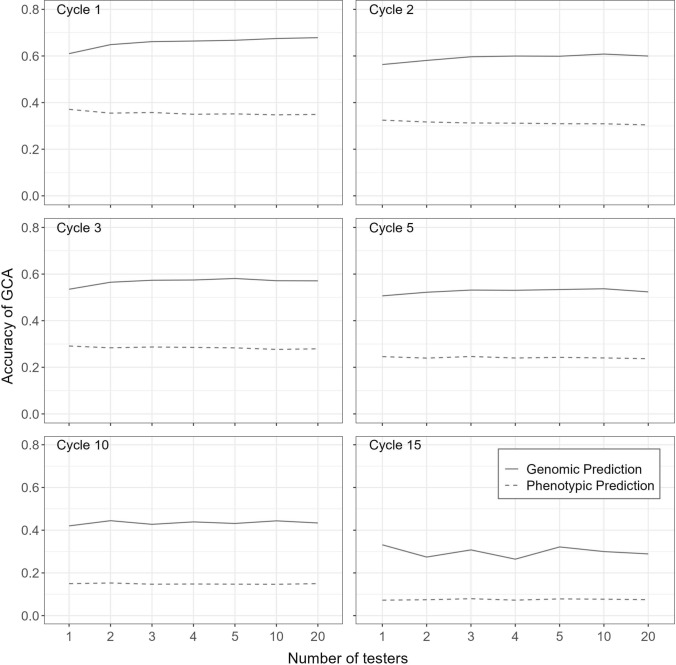
Prediction accuracy of genomic and phenotypic general combining ability (GCA) for the conventional single-tester testcross design and sparse testcrossing designs with 2, 3, 4, 5, 10, and 20 testers. Accuracies are shown for cycles 1, 2, 3, 5, 10, and 15 of the baseline breeding program under medium dominance degree and represent the mean prediction accuracies across 100 simulation replicates

All ten sparse testcrossing designs demonstrated higher genomic prediction accuracy for GCA than the conventional early-stage testcross design with a single tester. The gain in accuracy was largest during the early breeding cycles and declined as the genetic distance between pools increased. This is shown in Fig. 4, which plots genomic prediction accuracy of GCA under medium dominance degree for sparse testcrossing designs using 2, 3, 4, 5, 10, and 20 testers, alongside the conventional single-tester design, at cycles 1, 2, 3, 5, 10, and 15. Relative to phenotypic selection, genomic selection increased GCA accuracy by about 0.3 in cycle 1 and about 0.2 in cycle 15. Similar patterns were observed under high (Suppl. Fig. S7) and low (Suppl. Fig. S9) dominance degrees.

Figure 4 also shows that in early breeding cycles, prediction accuracy increased with the number of testers up to about 5–10 testers, after which it plateaued. For example, with 10 testers, accuracy increased by approximately 3%, 11%, and 23% for low, medium, and high dominance degrees, respectively, relative to the conventional single-tester strategy. Most of this gain was already captured with two or three testers. Because no further improvement was observed beyond 10 testers, results for designs with more than 20 testers under medium dominance are reported in Supplementary Fig. S8.

**Fig. 5:**
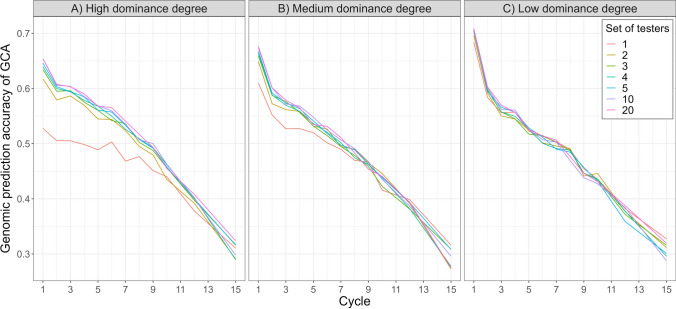
Genomic prediction accuracy of general combining ability (GCA) for the conventional single-tester testcross design and sparse testcrossing designs with 2, 3, 4, 5, 10 and 20 testers under **A** high, **B** medium and **C** low dominance degrees. Accuracies are shown for all 15 cycles of the baseline breeding program and represent the mean prediction accuracies across 100 simulation replicates

In contrast, by cycle 15, increasing the number of testers no longer improved prediction accuracy. This is shown in Fig. 5, which plots the trajectories of genomic GCA prediction accuracy across breeding cycles for the conventional early stage testcross design with one tester and the sparse testcrossing designs using 2, 3, 4, 5, 10, and 20 testers under low, medium, and high dominance degrees. Compared with Fig. 4, which emphasizes the effect of the number of testers, Fig. 5 highlights how the dominance degree affects genomic prediction accuracy over cycles. Gains in genomic prediction accuracy of GCA from sparse testcrossing were largest at high dominance degree; however, when dominance was low, the differences between sparse testcrossing designs and the conventional single-tester strategy were marginal.

Figures 4 and 5 also show a decline in prediction accuracy over time for all testcross designs, regardless of the simulated dominance degree. This decline can be attributed to a reduction in broad-sense heritability (Suppl. Fig. S5), caused by the decrease in hybrid genetic variance under selection (Suppl. Fig. S4) while the error variance remained constant.

### Hybrid genetic gain

**Fig. 6:**
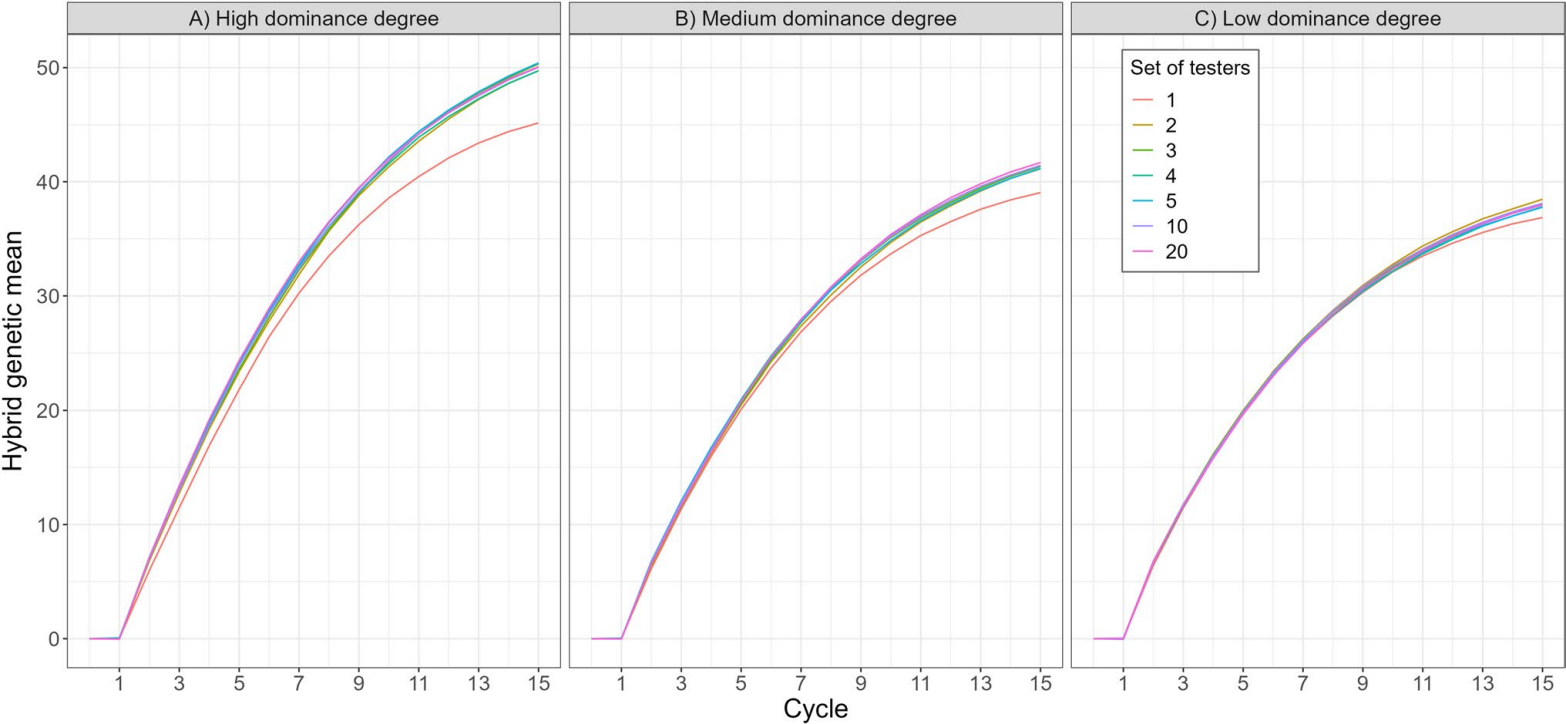
Hybrid genetic gain for the conventional single-tester testcross design and the sparse testcrossing designs with 2, 3, 4, 5, 10, and 20 testers under **A** high, **B** medium, and **C** low dominance degrees. Gains are shown for all 15 cycles of reciprocal recurrent genomic selection and represent mean values across 100 simulation replicates

Hybrid genetic gain was consistently higher when sparse testcrossing was used to predict genomic GCA than when the conventional early-stage testcross design with a single tester was used. This advantage was most pronounced under high dominance and diminished as the dominance degree decreased. This is shown in Fig. 6 which plots hybrid genetic gain across breeding cycles for the conventional single-tester design and the sparse testcrossing designs using 2, 3, 4, 5, 10 and 20 testers. For example, with 10 testers, hybrid genetic gain in cycle 15 was approximately 3% higher than with one tester under low dominance degree, 6% higher under medium dominance degree and 11% higher under high dominance degree.

Figure 6 also shows that genetic gain in cycle 15 increased with the number of testers, although differences among sparse testcrossing designs were relatively small. For example, under medium dominance degree, genetic gain was, on average, 5.3% higher with two testers than with one tester, but only 0.7% higher with ten testers than with two testers. Furthermore, using more than ten testers resulted in only marginal or no additional gain. This pattern is consistent with the plateau in prediction accuracy around five to ten testers observed in Fig. 4. As no further improvements were observed beyond ten testers, results for designs with more than 20 testers are shown in supplementary Fig. S10.

## Discussion

Sparse testcrossing using multiple testers can significantly increase genomic prediction accuracy of general combining ability (GCA) compared to conventional early-stage testcross designs using a single tester, thus enabling increased rates of hybrid genetic gain. To discuss these results, the discussion section is divided into two parts. In the first part, we explain that a relatively low number of three to five testers is sufficient for sparse testcrossing to be effective. We also emphasize that sparse testcrossing has the most significant impact on GCA prediction accuracy and hybrid genetic gain during the early stages of heterotic pool formation, particularly for traits where dominance is important. In the second part, we present how sparse testcrossing offers a simple and cost-efficient alternative to conventional testcross designs and incomplete factorial designs.

Overall, this simulation approach gives real-world hybrid breeding programs the opportunity to map their own level of heterotic pool differentiation onto our simulation results and draw conclusions about suitable numbers of testers.

### Sparse testcrossing increases GCA prediction accuracy and hybrid genetic gain 

All sparse testcrossing designs showed higher genomic prediction accuracy for GCA than the conventional testcross strategy with a single tester, which translated into increased hybrid genetic gain. This improvement is attributable to the use of a genomic relationship matrix (GRM) to predict each genotype’s GCA while employing multiple testers, thereby sampling the genetic diversity of the heterotic pool more comprehensively than a single tester without increasing testing resources.

In the early testing stages of hybrid breeding programs, large numbers of candidate lines must be evaluated for their GCA, which places substantial demands on testing resources. To efficiently use resources and maximize the number of lines evaluated, first-stage agronomic trials usually testcross each line with a single common tester. However, this single-tester design can lead to low GCA prediction accuracy, as it samples only a small fraction of the genetic variation present in the tester’s heterotic pool. Only as the number of genotypes decreases in later testing stages, multiple testers are used to more accurately reflect the diversity of haploblocks and their frequencies within their heterotic pool.

Sparse testcrossing enables the use of multiple testers in early-stage testing to predict the GCA of a genotype without increasing testing resource demands. By leveraging genomic relationships, sparse testcrossing effectively treats marker haplotypes as the primary unit of evaluation rather than the individual genotype. Although sparse testcrossing involves crossing each genotype with only a single tester, closely related genotypes with similar haplotypes are crossed with different testers. This enables the evaluation of individual haplotypes, as well as common haplotype combinations, for their value and complementarity against a broader, more representative set of haplotypes carried by various testers. By increasing GCA prediction accuracy, sparse testcrossing can also enable parent selection already after first stage of testing, thereby increasing genetic gain through more accurate GCA-based selection and shorter breeding cycles.

The effectiveness of sparse testcrossing, however, is tied to the use of genomic relationships. In the absence of genetic markers to leverage information from relatives, phenotypic selection showed substantially lower prediction accuracy than genomic prediction. As a result, sparse testcrossing without a GRM did not enhance prediction accuracy relative to using a single tester. In fact, crossing inbred lines with different testers can result in non-comparable GCA predictions and unfair comparisons. Therefore, sparse testcrossing without a GRM is not recommended.

### Sparse testcrossing enables enhanced GCA prediction accuracy with few testers

Genomic prediction accuracy of GCA and hybrid genetic gain increased with the number of testers but reached a plateau at around five to ten testers, with only minimal further increases in prediction accuracy beyond three testers. This suggests that using three to five testers for sparse testcrossing can effectively capture most of the diversity of haplotypes and haplotype combinations required to accurately predict the GCA of an inbred line from one heterotic pool when crossed with another heterotic pool.

Interestingly, increasing the number of testers beyond ten did not result in any further appreciable gains in prediction accuracy. While additional testers would be expected to increase accuracy by capturing more of the available haplotype diversity in the tester pool, no such improvement was observed. A plausible explanation is that a few cycles of crossing and selection in a closed breeding population are sufficient to build up strong linkage disequilibrium (Werner et al. 2023), leading to a substantial reduction of within-pool haplotype diversity as relatedness increases. As a result, a relatively small number of testers may adequately represent the most important haplotypes and haplotype combinations required for predicting GCA.

Additionally, there may be a trade-off between testing a greater diversity of haplotypes and haplotype combinations with the tester pool, and the replication rate of these combinations. While increasing the number of testers can enhance the representation of haplotype diversity, it comes at the cost of fewer observations per haplotype and thus lower accuracy of predicted effects, most notably for rare haplotypes that occur in only a limited set of testcross combinations. The advantage of sparse testcrossing in increasing prediction accuracy was most pronounced in the early cycles of crossing and selection, when haplotype diversity had not yet been reduced.

However, it is important to acknowledge that our simulations are simplified representations of complex biological systems. For example, all simulated genes (QTN) are biallelic, heterotic pools share the same set of loci with identical effects, and neither epistasis nor epigenetic effects were considered, as we lack a robust empirical basis for modeling realistic interaction and regulatory patterns. These simplifications are likely to underestimate the genetic diversity and genotype-specific background effects inherent to real-world breeding populations. With the genetic architecture of a quantitative trait in hybrid crops, such as yield, being more complex than in our simulation, GCA prediction accuracy may benefit slightly more from an increasing number of testers, and the benefit of increasing tester number from two to ten may be larger than the differences in accuracy and gain we report. However, without further empirical evidence, we consider three to five testers a practical compromise between resource efficiency, feasibility, and prediction accuracy.

### Sparse testcrossing enhances GCA prediction accuracy most when genetic distance between heterotic pools is low and diversity is high

Sparse testcrossing improved prediction accuracy and genetic gain most during the early breeding cycles, when genetic distance between pools was low to moderate and haplotype diversity within pools was high. This improvement diminished as the genetic distance between pools increased and genetic diversity within pools decreased, with one or a few testers becoming sufficient to represent the reduced diversity of haplotypes and haplotype combinations in the testcrosses.

At first glance, these results might suggest that sparse testcrossing is mainly relevant for emerging hybrid breeding programs, where germplasm has not yet split into well-defined heterotic groups or shows only modest divergence. However, we argue that this conclusion does not hold under the conditions of a practical breeding program. First, our simulations used a simplified, aggressive rapid cycle genomic selection strategy for population improvement, applied to a single quantitative trait, controlled by biallelic QTN, in a closed breeding population with no diversity management and no introgression of external material. In addition, the framework ignored genetic factors, such as genotype-by-environment (GxE) interaction, epistasis and mutation. Together, these assumptions are likely to substantially accelerate divergence and loss of diversity relative to real-world breeding programs. In practice, breeders select for many positively or negatively correlated traits across multiple environments, and selection is less efficient and accurate, so divergence between heterotic groups builds up more slowly. Second, one simulation cycle in our study may correspond to about four to ten years of selection in a commercial program, so even programs with three or four decades of breeding may still resemble the early simulation cycles. In fact, even long standing breeding programs may not have reached the level of high divergence between heterotic pools and low within pool diversity observed in the late simulation cycles.

For example, using publicly available data from the University of Hohenheim’s maize breeding program, Legarra et al. (2023) reported a Nei’s minimum genetic distance of 0.17, which fall within the early simulation cycles 3–4. Also, a recent study of a European hybrid dent maize breeding program managed by Limagrain Field Seeds estimated an average *F*_*ST*_ = 0.223 between SS and NSS heterotic groups after 4 decades (Kadoumi et al. 2025), which in our simulation would correspond approximately to cycle 2 (we computed *F*_*ST*_ but results are not shown). Therefore, not only emerging hybrid programs but also elite maize breeding programs may still benefit from implementing sparse testcrossing.

While developed in the context of inbred-hybrid breeding in maize, our findings provide general trends that are generally applicable to other diploid (or diploid-like) inbred-hybrid crops, including those with shorter hybrid breeding histories such as canola or sorghum, supporting a crop-agnostic interpretation of heterotic pool differentiation. For more accurate estimation of expected gains and cost–benefit outcomes, we recommend that breeding programs evaluate the potential benefit of sparse testcrossing through customized simulations using their own actual genotypic data and detailed program specific features.

### Sparse testcrossing enhances GCA prediction accuracy and hybrid genetic gain when dominance is high

Gains in genomic prediction accuracy of GCA from sparse testcrossing were largest at high dominance degree, whereas under low dominance the advantage over the conventional single-tester strategy was marginal. This suggests that, by employing multiple testers, sparse testcrossing more effectively captures the dominance genetic effects contributing to GCA that arise from complementary haplotype block interactions, thereby improving GCA prediction accuracy and ultimately translating into increased hybrid genetic gain.

The GCA effect involves both “biological” additive ($$a)$$ and dominance ($$d$$) effects, as well as the allele frequencies in the tester’s heterotic pool (Bernardo 2020; González-Diéguez et al. 2021). As shown in Eq. 3, the contribution of non-additive effects to the GCA depends on both the magnitude of dominance effects and the tester’s allele frequencies. When dominance is high, using the conventional testcross strategy with a single tester may poorly represent the haplotype diversity and allele frequencies in the tester pool, leading to low GCA prediction accuracy. In contrast, sparse testcrossing, through the use of multiple testers, more comprehensively samples the haplotype diversity and allele frequencies in the tester pool, making a better exploitation of beneficial interaction between complementary alleles and thereby improves GCA prediction accuracy.

When dominance is low, however, its contribution to the GCA is marginal, and the GCA is mainly driven by additive effects; consequently, accurate representation of haplotype diversity and allele frequencies in the tester pool becomes less critical. As a result, increasing the number of testers through sparse testcrossing provides little to no gain in GCA prediction accuracy.

### Sparse testcrossing preserves genetic diversity within pools and enhances long-term hybrid genetic gain

Sparse testcrossing led to a slower decline of genetic variance within heterotic pools, thereby generating hybrid genetic gain that was not only higher but also more sustainable than in the conventional single-tester design. This was because using several testers limited the tendency to select highly related genotypes as new parents.

When all candidate lines in early-stage testcross experiments are crossed to a single tester, lines with superior GCA often share a common genetic background, with similar haploblock combinations and interaction effects with that tester. As a result, selection based on GCA predicted with a single tester systematically favors closely related lines, for example from the same family (Suppl. Fig. S13, bottom row), and imposes a strong selection bottleneck on early-stage genetic variation, unless kinship is actively managed. This bottleneck accelerates the loss of genetic diversity within pools (Suppl. Fig. S13, Jaccard coefficient at the top row; Suppl. Fig. S11, fraction of fixed alleles at bottom row), and ultimately limits further genetic gain. Particularly when genomic selection is applied, the combination of increased accuracy and rapid parent recycling in a closed breeding program can further exacerbate the erosion of genetic diversity within pools.

Sparse testcrossing effectively shifted selection pressure from closely related genotypes that performed well with the same single tester toward a genetically more diverse set of inbred lines with high GCA predicted from multiple testers. However, because the slower reduction in genetic variance was a byproduct of sparse testcrossing rather than an explicit breeding objective, we recommend combining it with an active strategy to manage genetic variance, such as optimum contribution selection (Woolliams et al. 2015).

### 4.5 Sparse testcrossing offers an effective balance between simplicity, increased prediction accuracy and resource efficiency compared to incomplete factorial designs

By combining simple testcross designs with efficient use of resources and higher prediction accuracy for GCA, sparse testcrossing offers a practical alternative to conventional testcrossing strategies, in which each candidate is crossed to the same tester(s), and to incomplete factorial designs that randomly sample testcrosses from a full factorial mating design (Seye et al. 2020; Lorenzi et al. 2022).

Incomplete factorial designs use a large number of unevaluated candidate lines from each pool as testers, under the assumption that this provides a better representation of the testers’ heterotic pools and thereby increases GCA prediction accuracy. However, although incomplete factorial designs can offer the advantage of evaluating twice as many lines for a given number of testcrosses or requiring half the phenotyping effort for the same number of candidates (Lorenzi et al. 2022), their implementation poses significant logistical challenges primarily due to labor-intensive and costly manual pollination required to achieve the intended hybrid combinations (Seye et al. 2020; Lorenzi et al. 2022), further intensified by the need to synchronize flowering between the two pools and produce sufficient seed for multi-environment evaluations, making it an impractical solution for breeding programs.

To compare our sparse testcrossing designs to an incomplete factorial testcross design, we simulated a sparse testcross design in which all 1,200 from both heterotic pools were randomly crossed with each other to generate 1,200 hybrids, approximating an incomplete factorial. We observed that GCA prediction accuracies were similar to those achieved with sparse testcrossing designs using three to five testers (Suppl. Figs S7, S8 and S9 for high, medium and low dominance degrees), indicating that a few testers can provide a sufficiently good representation of the testers’ heterotic pool with substantially less logistical effort.

In our simulations, the sparse testcrossing designs were compared with a conventional single-tester design that already applied genomic selection. For simplicity, we assumed equal phenotyping and genotyping resources for testing, ignoring the additional cost of more isolation blocks or hand pollinations required to produce testcross seed. In practice, these costs will depend on the number of testers and the seed production strategy. For example, using five testers will require ten (5 testers × 2 pools) isolated blocks which indeed entails additional logistical effort and cost. In such cases, hand pollination is likely the more practical option. It is noteworthy that in many public and small hybrid breeding programs breeders use single-cross testers as female components of three-way hybrids to ensure adequate testcross seed production, and thus routinely perform hand pollination. Implementing sparse testcrossing with few testers through hand pollination would therefore involve only a slight increase in logistics, but significantly simpler than managing an incomplete factorial design.

### Representing heterotic pools: choice of testers in simulation versus practice

In our simulations, new testers were drawn at random in every cycle so that comparisons among testcross designs were not tied to any specific tester selection strategy. Across the 100 simulation replicates, this sampling strategy produced tester sets that, on average, accurately represented the allele frequencies of the tester pool. This provided robust estimates of prediction accuracy that can be attributed to the testcross designs rather than the way testers were chosen. We, therefore, consider random tester selection the most suitable choice for comparing testcross strategies in simulation, while acknowledging that it has limited direct relevance for real-world breeding programs, where tester selection is strongly shaped by practical and logistical constraints additional to theoretical considerations.

In practice, testers are rarely chosen at random. They are typically elite lines that already serve as parents of commercial hybrids and are selected for high GCA, strong per se performance and favorable pollen traits, which are critical for reliable commercial seed production (Melchinger and Frisch 2023). Furthermore, in various public programs, such as the CIMMYT maize breeding programs in Eastern and Southern Africa, single-cross testers used as female parents of three-way hybrids are specifically selected for seed production traits.

Although these criteria facilitate robust testcross performance and early discovery of promising hybrids, they restrict tester choice to former breeding cycles. As a result, the testers tend to reflect allele frequencies of past generations rather than the current haplotype diversity of the heterotic pool, which can compromise the accuracy of GCA prediction with respect to the present tester pool. In fact, classical theoretical and experimental work indicates that low-performing testers with low frequencies of favorable alleles can be more informative for improving GCA than elite testers, because they more effectively indicate which lines from the other pool carry superior haplotype combinations to complement the unfavorable and detrimental alleles in the tester pool (Hull 1945; Rawlings and Thompson 1962; Hallauer et al. 2010).While tester selection should ideally balance theoretical considerations with practical constraints and simplicity, working out such rules lies beyond the scope of this simulation study.

## Supplementary Information

Below is the link to the electronic supplementary material.

Supplementary file1 (DOCX 2014 KB)

## Data Availability

Not applicable.

## Code availability

All simulations were done using AlphaSimR version 1.4.2 (Chris Gaynor et al. 2021). The R-scripts used to generate the results presented in this simulation study are available on the GitHub repository at https://github.com/dogrdc/Sparse-testcrossing.
