# Supplementary material for: Sparse testcrossing for early-stage genomic prediction of general combining ability to increase genetic gain in maize hybrid breeding programs
Source: Theor Appl Genet. 2026 Feb 24;139(3):78. doi: 10.1007/s00122-026-05169-x (PMC12932368; doi:10.1007/s00122-026-05169-x)
Supplement: Supplementary file 1 — Supplementary file1 (DOCX 2014 KB) [file 122_2026_5169_MOESM1_ESM.docx]

**Supplemental material**

**Theoretical and Applied Genetics**

**Sparse testcrossing for early-stage genomic prediction of general combining ability to increase genetic gain in maize hybrid breeding programs**

David O. González-Diéguez^1,2^, Gary N. Atlin^5^, Yoseph Beyene^3^, Dagne Wegary^4^, Dorcus C. Gemenet^1,3^, Christian R. Werner^1,2^

^1^Breeding Innovation and Modernization, Consultative Group of International Agricultural Research (CGIAR), Texcoco, Mexico.

^2^ International Maize and Wheat Improvement Center (CIMMYT), Texcoco, Mexico.

^3^ International Maize and Wheat Improvement Center (CIMMYT), Nairobi, Kenya.

^4^International Maize and Wheat Improvement Center (CIMMYT), Harare, Zimbabwe.

^5^ Bill & Melinda Gates Foundation, Seattle, WA 98109, USA

**Corresponding author:**

David O. González-Diéguez

International Maize and Wheat Improvement Center (CIMMYT), Km 45, Carretera Mexico-Veracruz, Texcoco 56237, Edo. de México, Mexico

Tel : +52 5558042004 ext. 2146

E-mail: D.G.DIEGUEZ@cgiar.org


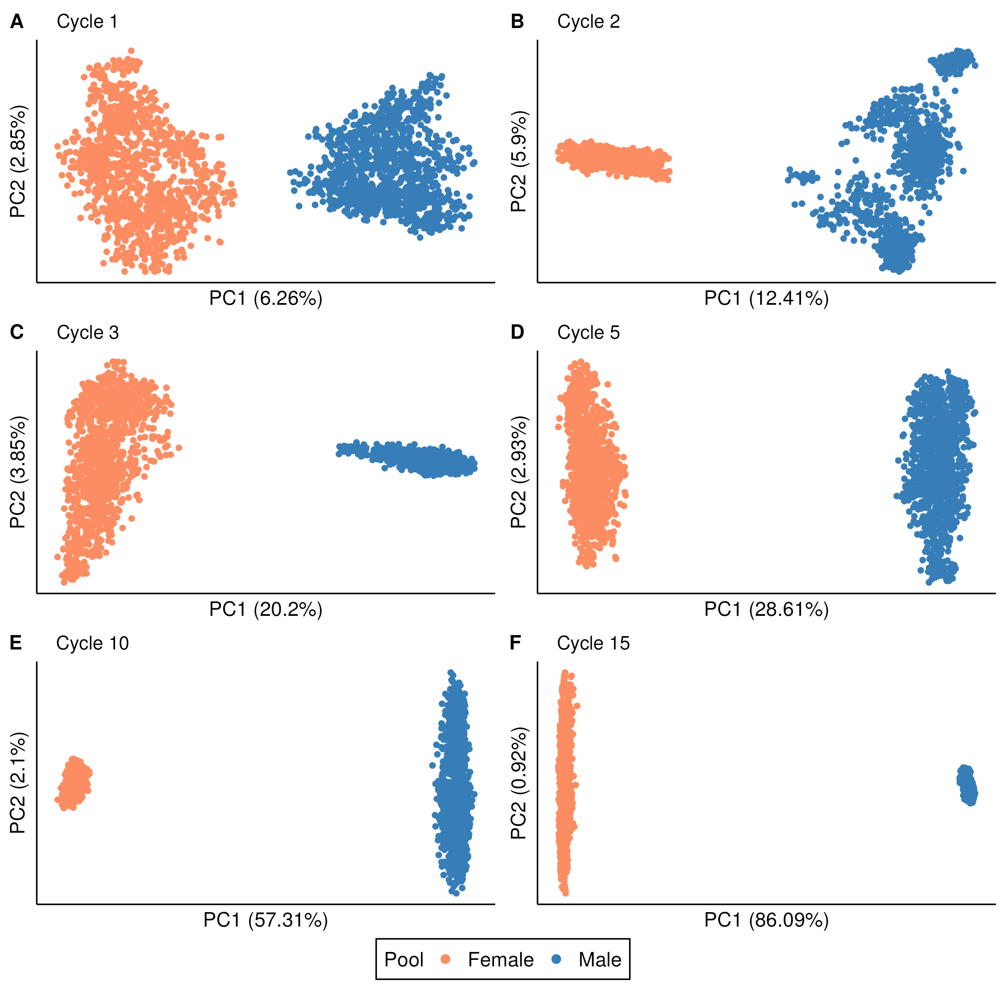
**Fig. S1** Principal component analysis (PCA) of the genomic relationship matrix of the two heterotic pools in the baseline breeding program at cycles 1, 2, 3, 5, 10, and 15. Orange and blue points represent the female and male heterotic pools, respectively. The first and second principal components (PC1 and PC2) are shown on the x and y axes, respectively. The proportion of variance explained by PC1 and PC2 is given in parentheses. Results shown correspond to a single, randomly selected simulation replicate of the baseline breeding program under low dominance degree.


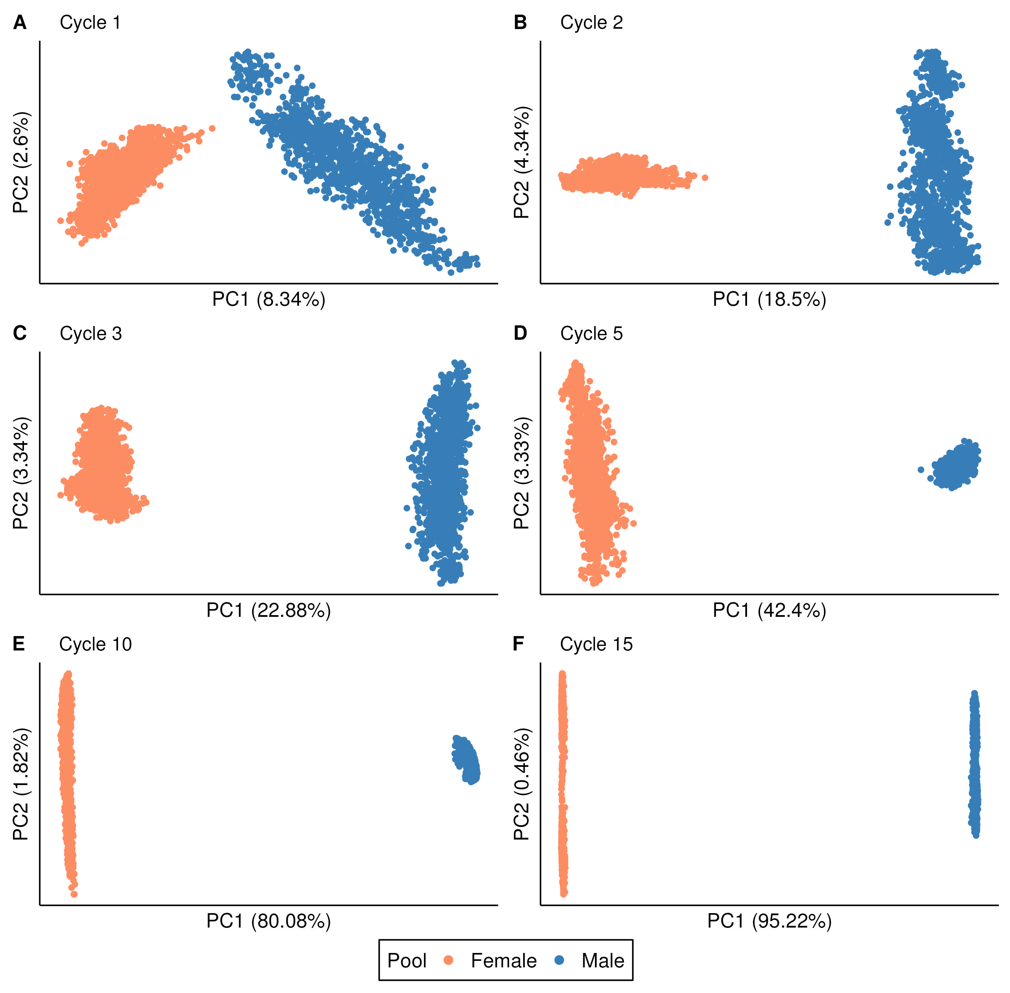
**Fig. S2** Principal component analysis (PCA) of the genomic relationship matrix of the two heterotic pools in the baseline breeding program at cycles 1, 2, 3, 5, 10, and 15. Orange and blue points represent the female and male heterotic pools, respectively. The first and second principal components (PC1 and PC2) are shown on the x and y axes, respectively. The proportion of variance explained by PC1 and PC2 is given in parentheses. Results shown correspond to a single, randomly selected simulation replicate of the baseline breeding program under high dominance degree.


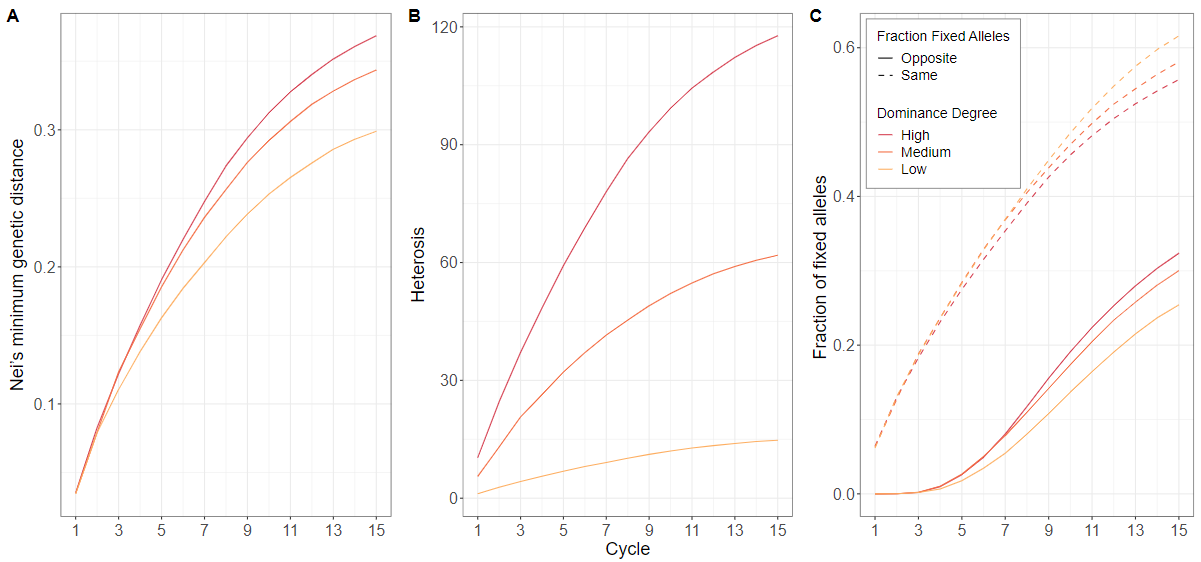


**Fig. S3** Metrics of genetic divergence between heterotic pools over 15 cycles of GCA-based selection in the baseline reciprocal recurrent genomic selection breeding program for high, medium and low degrees of dominance. A) Mean Nei’s minimum genetic distance, B) mean heterosis, and C) mean fraction of fixed (same or opposite) alleles in the two pools. The results are averaged across all 100 simulation replications of the baseline breeding program.


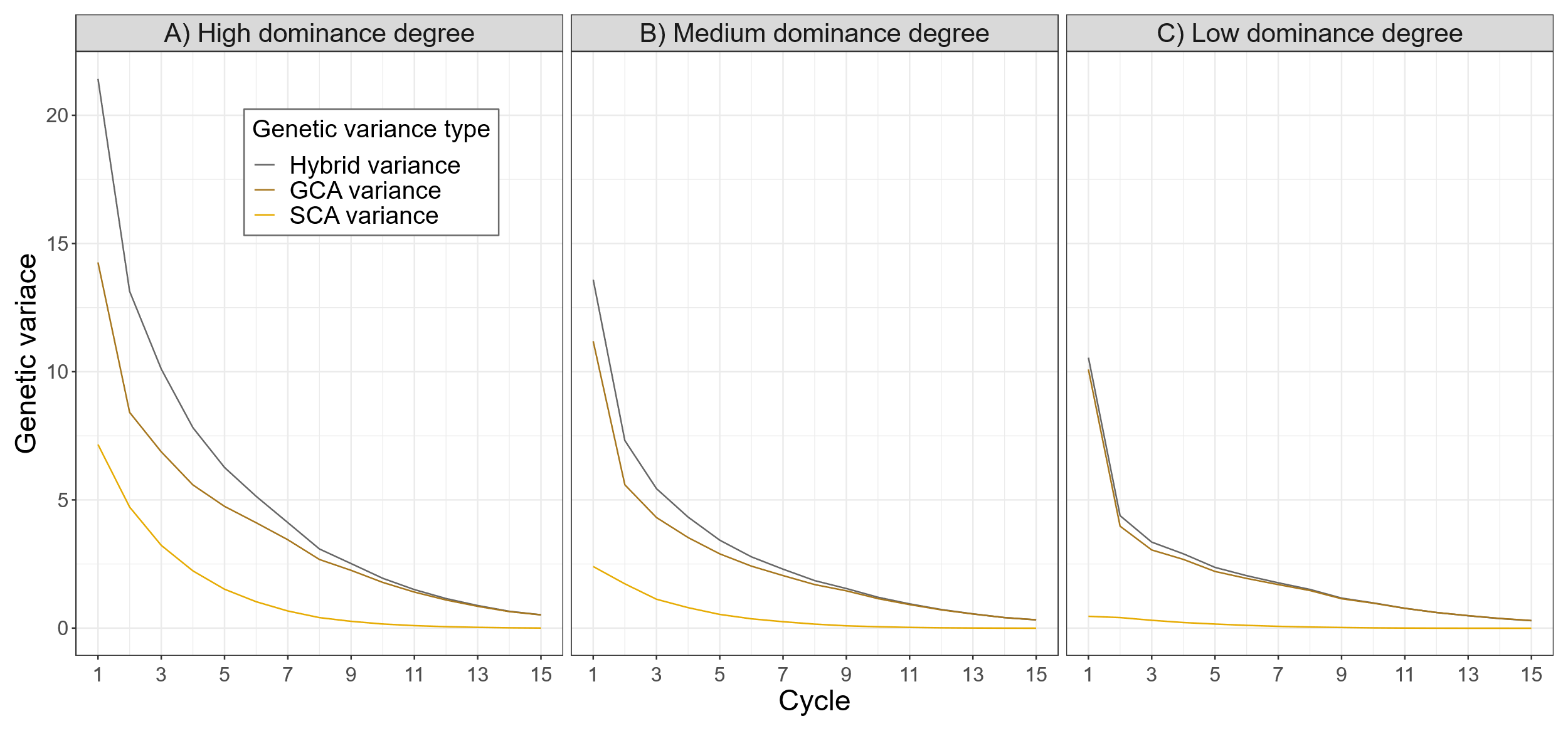


**Fig. S4** Hybrid genetic variance, GCA variance, and SCA variance monitored across 15 cycles of selection in the baseline reciprocal recurrent reciprocal genomic selection breeding program, for A) high, B) medium and D) low degrees of dominance. The results are averaged across all 100 simulation replications of the baseline breeding program.

**
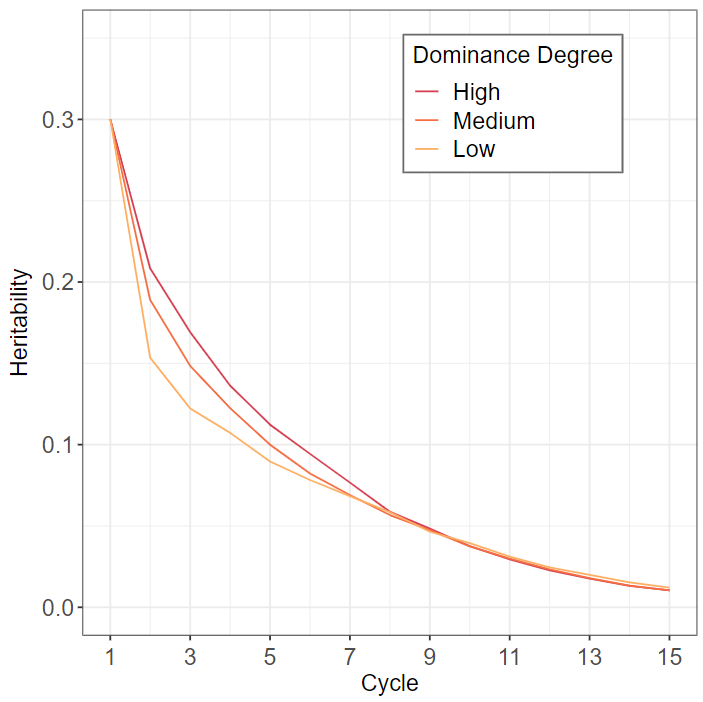
**

**Fig. S5** Broad-sense heritability ($H^{2}$) monitored across 15 cycles of selection in the baseline recurrent reciprocal genomic selection breeding program, for A) high, B) medium and D) low dominance degrees. Results are averaged across all 100 simulation replications of the baseline breeding program.


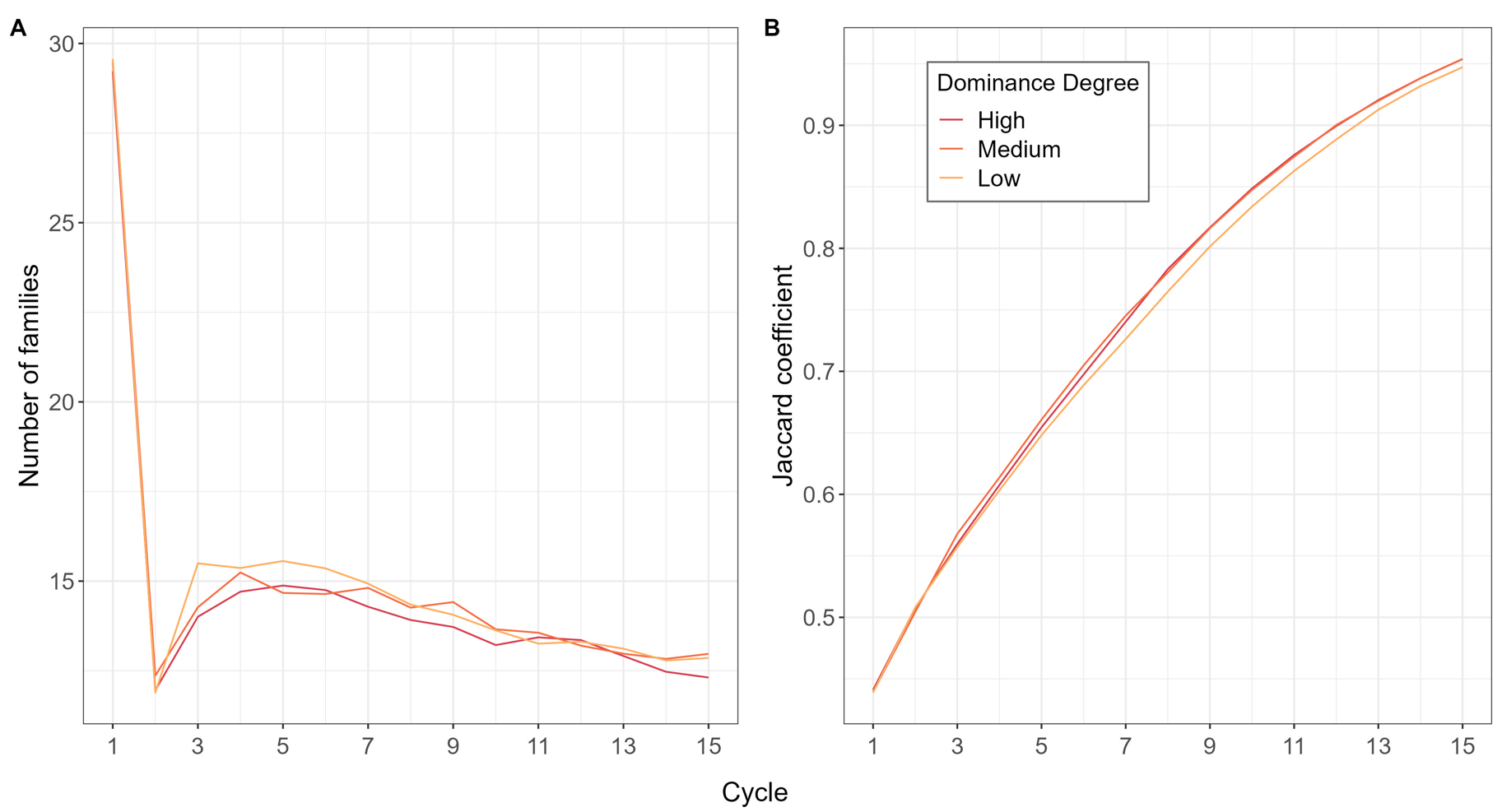


**Fig. S6** Metrics of genetic diversity within the breeding pools monitored across 15 cycles of selection in the baseline recurrent reciprocal genomic selection breeding program for high, medium and low dominance degrees, averaged across all 100 simulation replications of the baseline breeding program. A) The number of families of selected parents averaged across both female and male heterotic pools, initially increased during the early selection cycles (2–5) before exhibiting a subsequent decline. B) Jaccard similarity coefficient averaged across female and male heterotic pools, shows a faster increase indicating that genetic diversity within pools decreased progressively as a consequence of selection. The Jaccard similarity coefficient (Jaccard 1908) was calculated using SNP marker genotypes of selected inbred parents within each pool. A similarity matrix was generated using the *vegdist()* function from the vegan R package (Oksanen et al. 2001). The average value of the lower triangular portion of this matrix was then used as the final similarity metric.


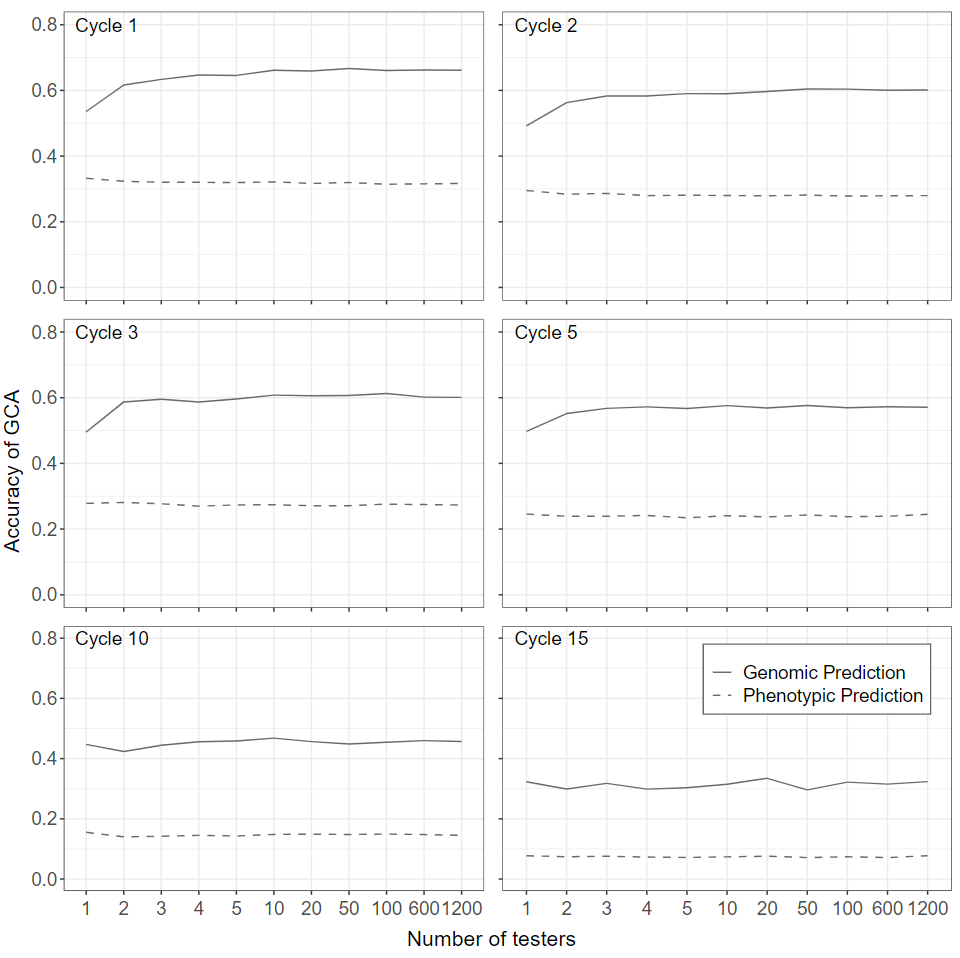


**Fig. S7** Prediction accuracy of genomic and phenotypic general combining ability (GCA) for the conventional single-tester testcross design and sparse testcrossing designs with 2, 3, 4, 5, 10, 20, 50, 100, 600, and 1,200 testers. Accuracies are shown for cycles 1, 2, 3, 5, 10, and 15 of the baseline breeding program under high dominance degree and represent the mean prediction accuracies across 100 simulation replicates.

**
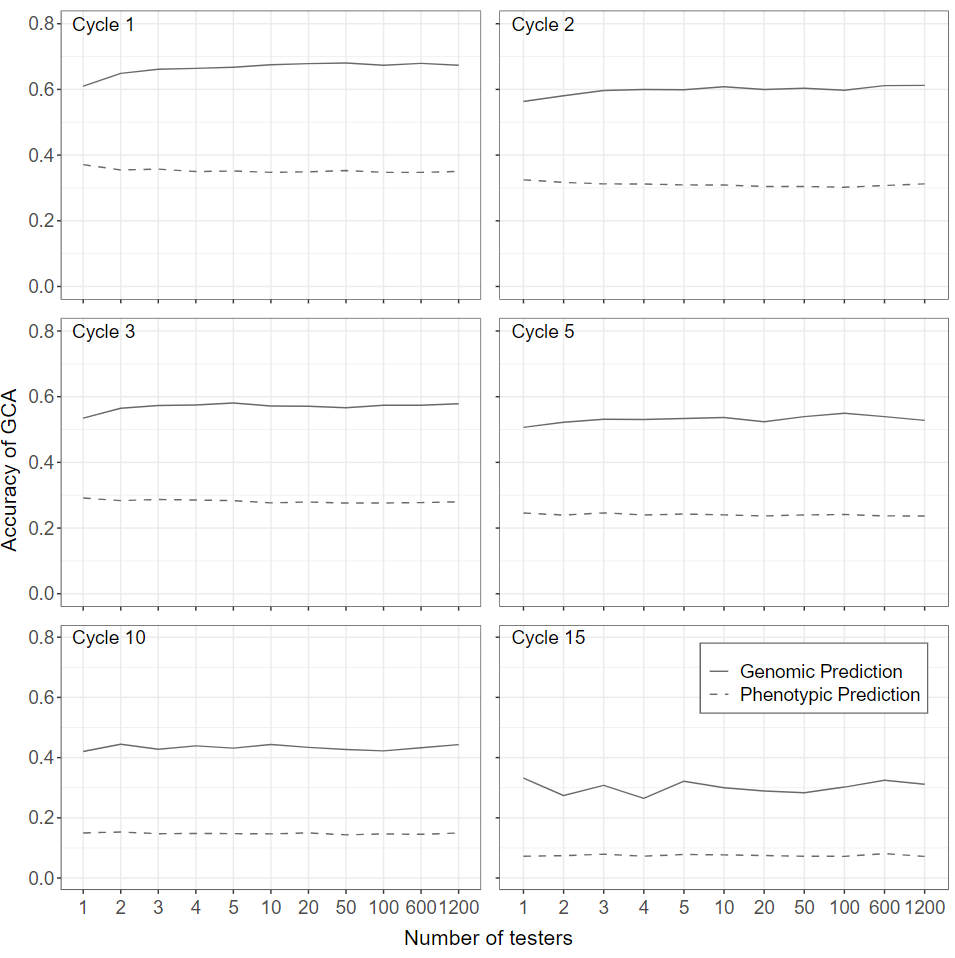
**

**Fig. S8** Prediction accuracy of genomic and phenotypic general combining ability (GCA) for the conventional single-tester testcross design and sparse testcrossing designs with 2, 3, 4, 5, 10, 20, 50, 100, 600, and 1,200 testers. Accuracies are shown for cycles 1, 2, 3, 5, 10, and 15 of the baseline breeding program under medium dominance degree and represent the mean prediction accuracies across 100 simulation replicates.


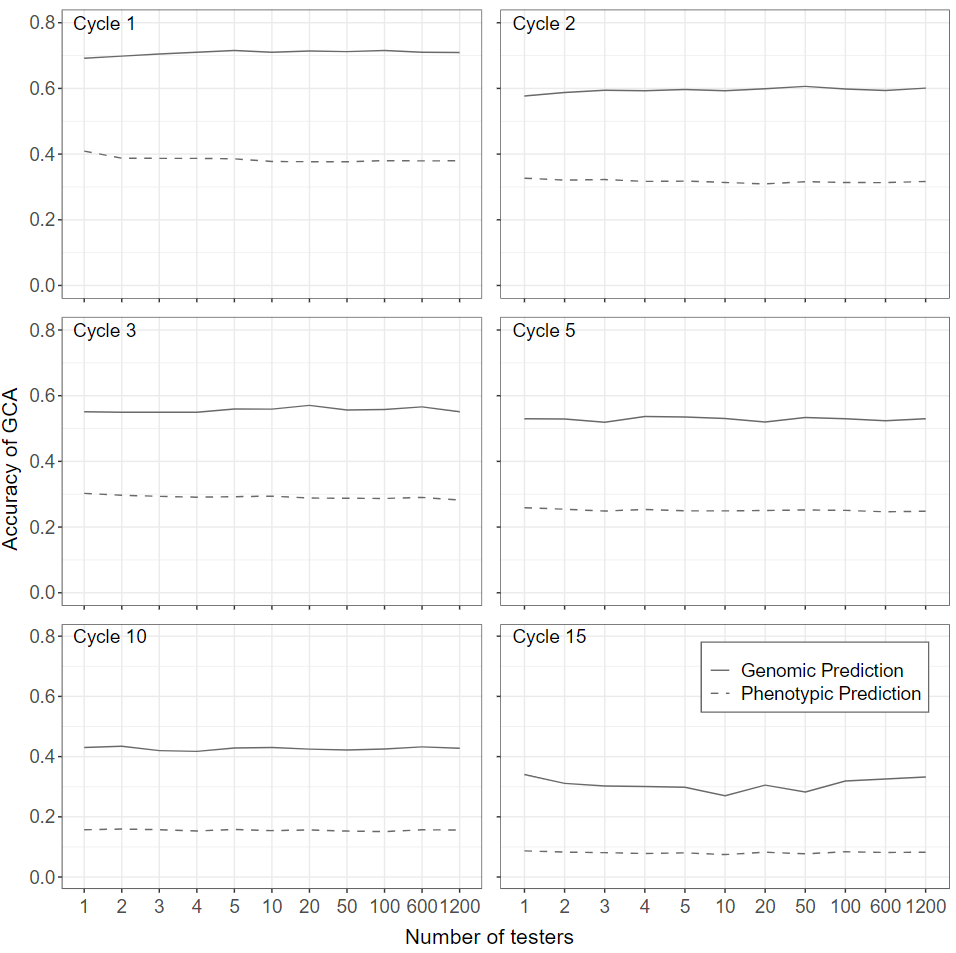


**Fig. S9** Prediction accuracy of genomic and phenotypic general combining ability (GCA) for the conventional single-tester testcross design and sparse testcrossing designs with 2, 3, 4, 5, 10, 20, 50, 100, 600, and 1,200 testers. Accuracies are shown for cycles 1, 2, 3, 5, 10, and 15 of the baseline breeding program under low dominance degree and represent the mean prediction accuracies across 100 simulation replicates.


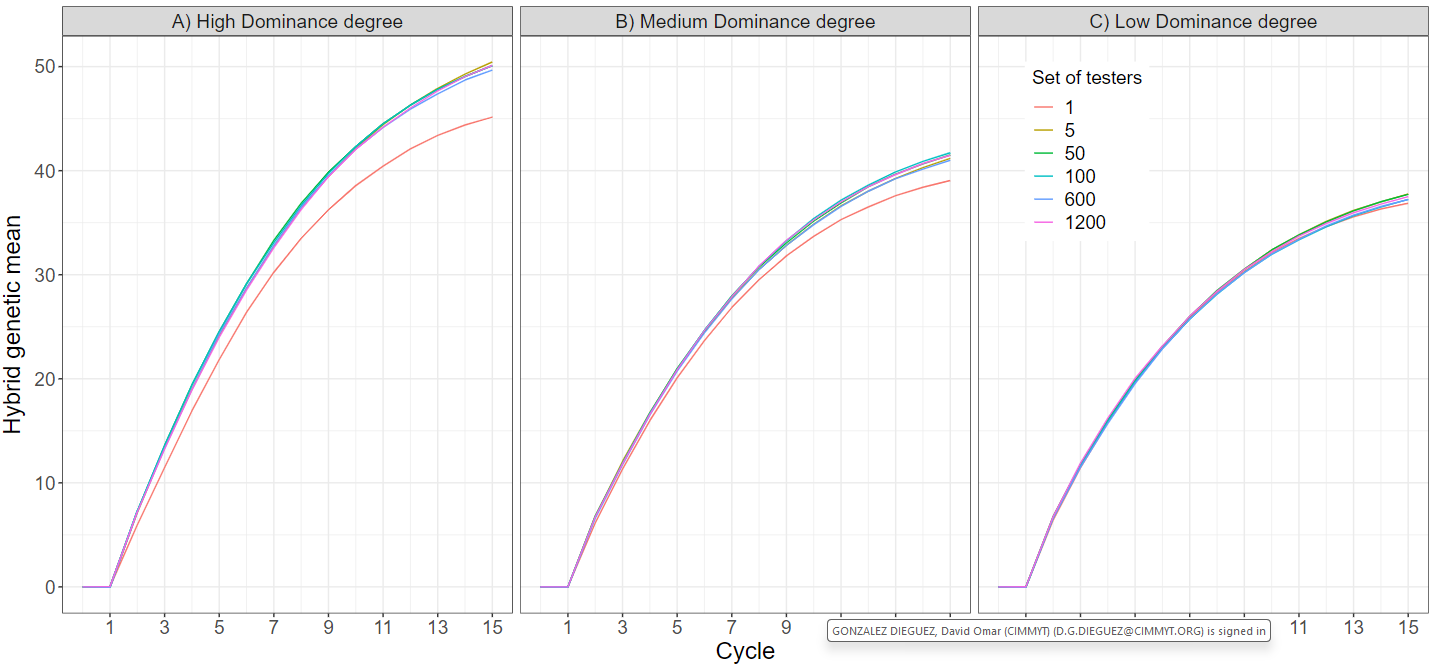


**Fig. S10** Hybrid genetic gain for the conventional single-tester testcross design and the sparse testcrossing designs with 5, 50, 100, 600 and 1200 testers under A) high, B) medium, and C) low dominance degrees. Gains are shown for all 15 cycles of reciprocal recurrent genomic selection and represent mean values across 100 simulation replicates.

**
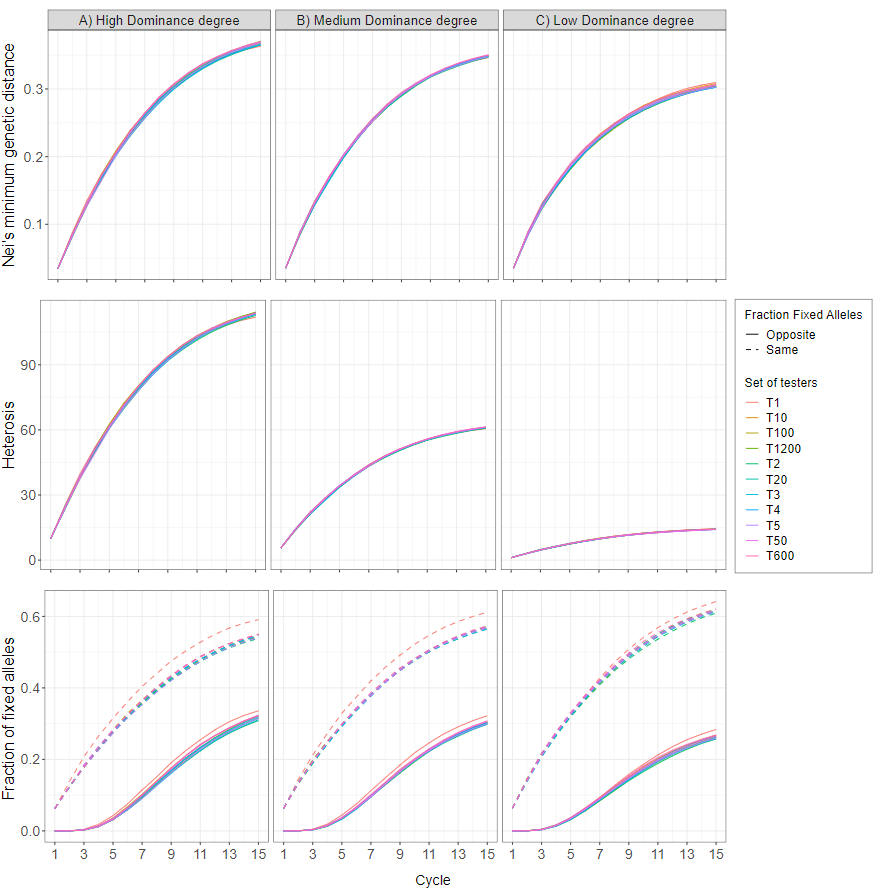
Fig. S11** Metrics of genetic divergence between heterotic pools monitored across 15 cycles of selection for sparse testcross designs with sets of 2, 3, 4, 5, 10, 20, 50, 100, 600 and 1200 testers, and the conventional testcross design with a single-tester. Mean Nei’s minimum genetic distance between heterotic pools, mean heterosis and mean fraction of fixed (same and opposite) alleles in the two heterotic pools, for A) high, B) medium and c) low dominance degrees. Results are averaged across all 100 simulation replications.

**
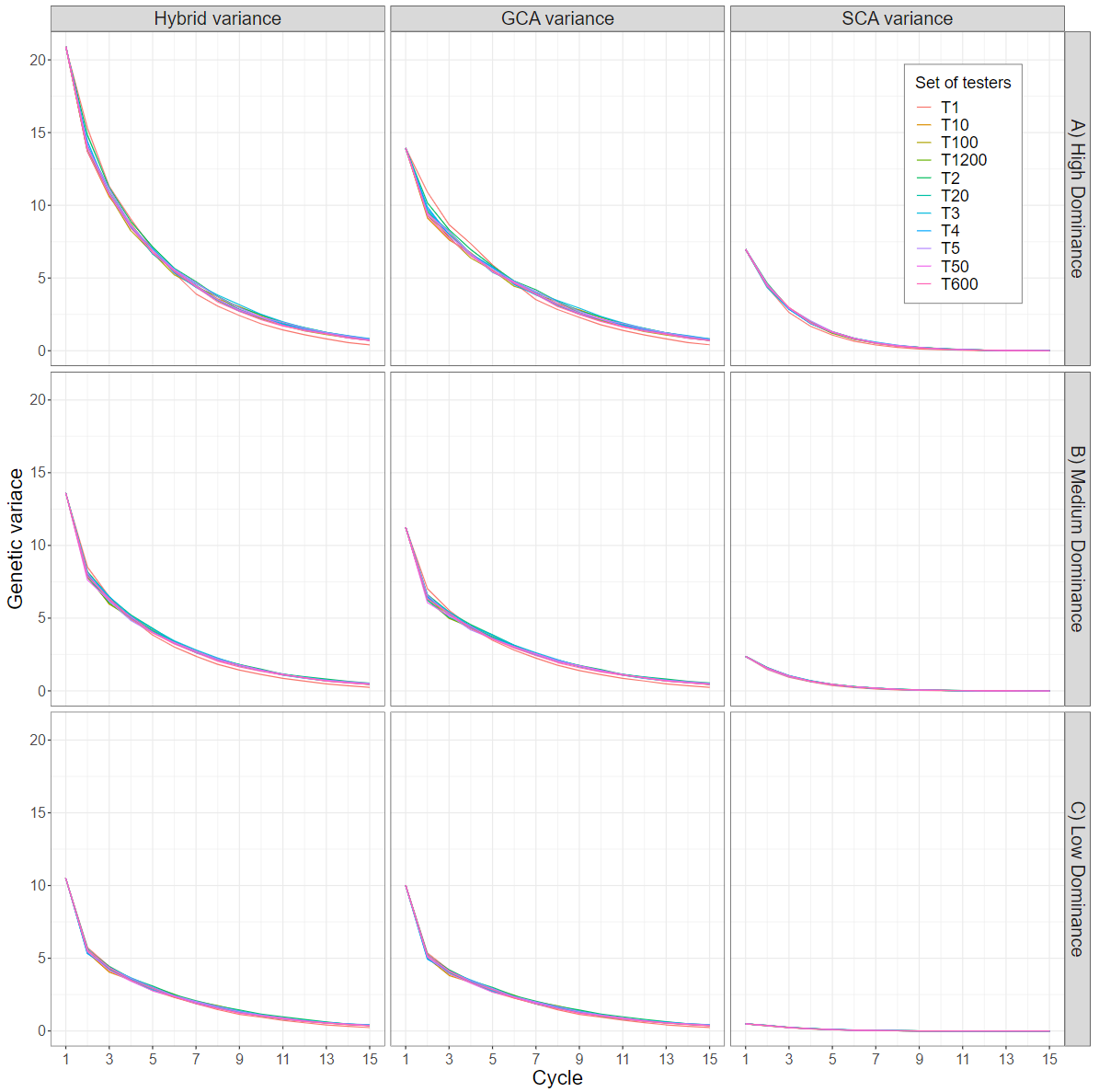
**

**Fig. S12** Total genetic variance of hybrids, GCA variance of inbred lines, and SCA variance in hybrids observed by using sparse testcross designs with sets of 2, 3, 4, 5, 10, 20, 50, 100, 600 and 1200 testers, compared to a conventional early-stage testcross design with a single-tester, across 15 cycles of recurrent reciprocal genomic selection and for A) high, B) medium and C) low dominance degrees. Results are averaged across all 100 simulation replications.

**
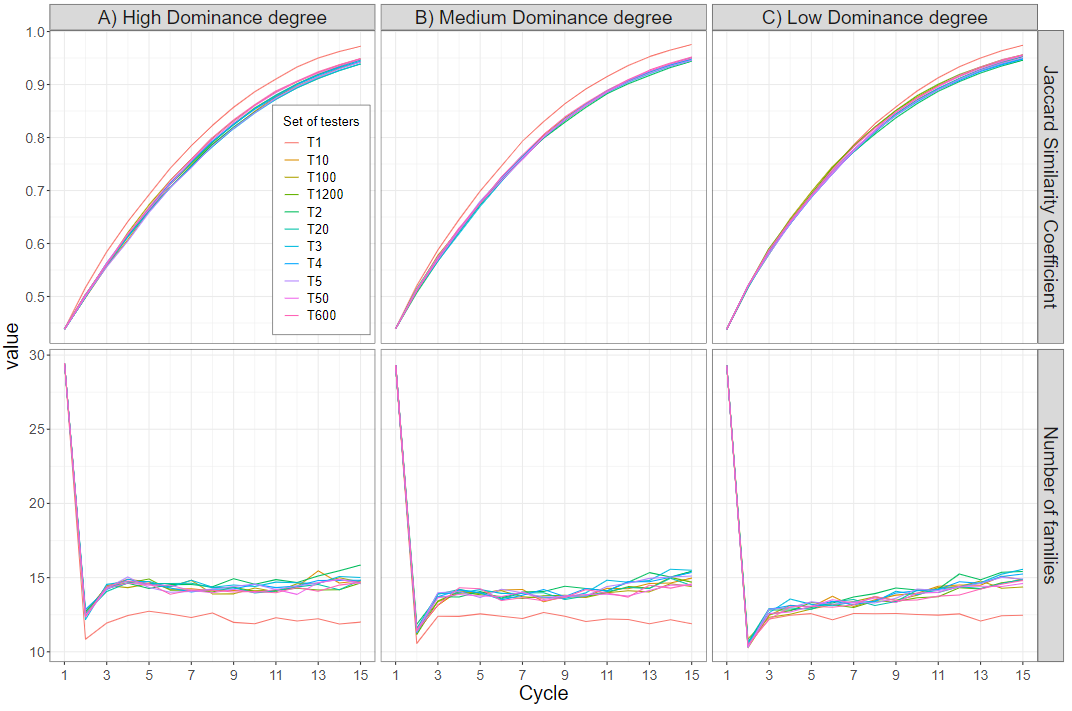
**

**Fig. S13** Metrics of genetic diversity within the breeding pools monitored across 15 cycles of selection for sparse testcross designs with sets of 2, 3, 4, 5, 10, 20, 50, 100, 600 and 1200 testers, and the conventional testcross design with a single-tester. Mean number of families of selected parents (bottom row) averaged across both female and male heterotic pools, and within pool Jaccard similarity coefficient (top row) averaged across male and female pools, for A) high, B) medium and c) low dominance degrees. Results are averaged across all 100 simulation replications. The Jaccard similarity coefficient (Jaccard 1908) was calculated using SNP marker genotypes of selected inbred parents within each pool. A similarity matrix was generated using the *vegdist()* function from the vegan R package (Oksanen et al. 2001). The average value of the lower triangular portion of this matrix was then used as the final similarity metric. The number of families within pools was lower with a single tester than with sparse testcrossing designs and declined slightly over time, whereas it increased under sparse testcrossing. The conventional testcross strategy showed a lower within-pool genetic diversity as indicated by a faster increase in the Jaccard Similarity Coefficient.

**
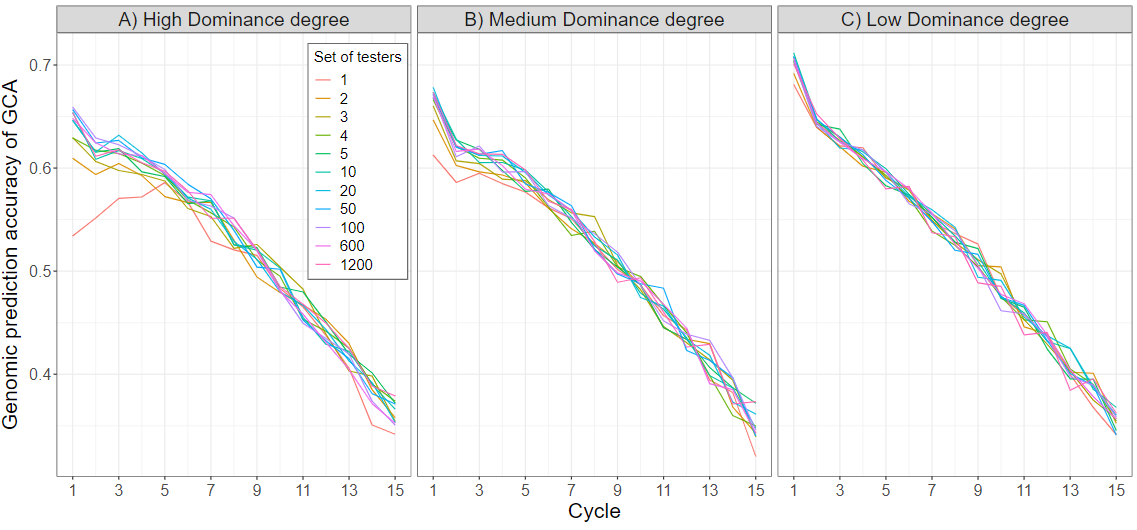
**

**Fig. S14** Genomic prediction accuracy of genomic combining ability (GCA) for the conventional single-tester testcross design and sparse testcrossing designs with 2, 3, 4, 5, 10, 20, 50, 100, 600 and 1200 testers under A) high, B) medium and C) low dominance degrees. Accuracies are shown for all 15 cycles of the baseline breeding program and represent the mean prediction accuracies across 100 simulation replicates.

**Literature cited**

Jaccard P (1908) Nouvelles recherches sur la distribution florale [New research on floral distribution]. Bulletin de la Societe Vaudoise des Sciences Naturelles 44:

Oksanen J, Simpson GL, Blanchet FG, et al (2001) vegan: Community Ecology Package. CRAN: Contributed Packages
